# Eco-evolutionary dynamics of massive, parallel bacteriophage outbreaks in compost communities

**DOI:** 10.1126/sciadv.aeb8246

**Published:** 2026-05-29

**Authors:** Jeroen Meijer, Petros Skiadas, Paul B. Rainey, Paulien Hogeweg, Bas E. Dutilh

**Affiliations:** ^1^ Theoretical Biology and Bioinformatics, Science for Life, Utrecht University, Padualaan 8, 3584 CH, Utrecht, the Netherlands.; ^2^ Institute of Biodiversity, Ecology, and Evolution, Faculty of Biological Sciences, Cluster of Excellence Balance of the Microverse, Friedrich Schiller University Jena, 07743, Jena, Germany.; ^3^ Department of Microbial Population Biology, Max Planck Institute for Evolutionary Biology, Plön, Germany.; ^4^ Laboratory of Biophysics and Evolution, CBI, ESPCI Paris, Université PSL, CNRS, Paris, France.

## Abstract

Bacteriophages play critical roles in microbial ecosystems, yet their dynamics in complex natural communities remain poorly understood compared to simplified laboratory systems. Here, we tracked viral dynamics in 20 compost-derived microbial communities over 1 year. Communities formed two alternative stable types, each dominated by distinct cellulose degraders and comprising hundreds of genera. In one community type, we observed massive, parallel outbreaks of Theomophage, a previously uncharacterized member of the Schitoviridae, reaching up to 74% of metagenomic reads—the largest bacteriophage outbreak documented to date. Despite extensive replication, Theomophage displayed notable genetic stability during outbreaks and over time. In contrast, the experimental migration of viral communities triggered rapid evolution driven by recombination and the accumulation of newly arising mutations, particularly after colonization of communities of the alternative type in which the phage was initially absent. These results reveal the spatial and temporal scales at which bacteriophage microdiversity evolves in complex ecosystems and show that viral mixing, likely common in nature, can rapidly accelerate phage evolution.

## Teaser

A single bacteriophage can dominate microbial communities yet only evolves when migration changes its ecological context.

## INTRODUCTION

Viruses that infect bacteria (bacteriophages or phages) are ubiquitous and ecologically influential components of all microbial ecosystems, from oceans and soils to host-associated microbiomes. They regulate bacterial populations, drive nutrient cycling, and shape bacterial evolution by facilitating horizontal gene transfer (HGT) (*1*). Phages may also shape community structure and function, although evidence is mixed (*2*). These ecological and evolutionary effects arise from highly specific interactions between phages and their bacterial hosts, which are typically shaped by antagonistic coevolution (*3*–*6*). Identifying the biotic and abiotic factors that govern phage eco-evolutionary dynamics is essential for predicting microbial community diversity, stability, and function and for guiding applied interventions such as bacteriophage therapy (*7*).

Current knowledge of phage ecology and evolution is largely shaped by two contrasting study designs: simplified in vitro experimental systems in nutrient-rich media, typically consisting of one or a few phage-host pairs, and observational surveys of complex natural ecosystems using metagenomics or large-scale isolation and phenotyping (*8*–*10*). The ecological and evolutionary patterns observed with these two approaches often do not align (*9*–*15*). For example, experiments with single phage-host pairs show rapid coevolution proceeding via a series of selective sweeps, with bacteria evolving resistance through receptor modification or loss, and phage counteradaptation occurring through compensatory changes in phage tail proteins (*4*, *5*, *16*). In contrast, studies of natural ecosystems often reveal patterns of microdiversity suggesting long-term coexistence of resistant and susceptible hosts (*17*, *18*) and a notable genomic stability of phages (*19*, *20*). They also show that bacterial resistance in natural systems is shaped more by variation in defense systems than by receptor evolution (*3*, *21*, *22*). These differences indicate that selective pressures in simplified laboratory conditions differ fundamentally from those in complex natural environments. More broadly, our ability to extrapolate from laboratory models to predict viral dynamics in complex environments remains limited (*9*–*11*), prompting calls for experimental models that incorporate greater ecological complexity while retaining experimental tractability (*8*–*10*, *23*, *24*).

Several features of natural ecosystems likely contribute to these differences (*9*, *10*, *23*, *24*). Natural communities harbor extensive macro- (taxonomic) and micro- (strain-level) diversity, in contrast to the isogenic populations typical of laboratory experiments. In diverse communities, genetic novelty arises through not only mutation but also recombination and HGT. Increasing macrodiversity beyond a single phage-host pair can change both ecological and evolutionary trajectories [recently reviewed in (*9*, *24*)]. Natural ecosystems are also spatially heterogeneous, comprising fragmented patches (e.g., skin pores, gut crypts, marine snow, and soil particles) connected by restricted dispersal, which produces local populations linked intermittently by migration (*25*). These metapopulation dynamics are inherently different from those in homogeneous laboratory systems (*26*–*28*).

Microbial communities derived from natural ecosystems and propagated under laboratory conditions provide a promising middle ground between simplified laboratory systems and the full complexity of natural environments (*29*–*33*). When grown on complex carbohydrates, they maintain high taxonomic and functional diversity while remaining amenable to longitudinal sampling and controlled perturbation. These systems have been used to investigate bacterial community assembly and function (*29*–*33*), but applications to viruses remain limited (*34*, *35*). Here, we used time series data from compost-derived microbial communities grown on cellulose paper propagated under different regimes of viral migration (*36*) to investigate how standing microdiversity, bacteriophage migration, and community context shape bacteriophage eco-evolutionary dynamics. Same as other biopolymer-degrading microbial communities, cellulose-based communities form trophic cascades in which primary degraders supply nutrients for downstream consumers (*30*, *32*), capturing key ecological interactions of natural microbial ecosystems. In this realistic yet tractable ecological system, we used genotype-resolved viromics to show that bacteriophage evolution can shift from long-term stasis to rapid change when migration introduces viruses into new community contexts.

## RESULTS

### Compost communities cluster in two distinct and stable community types

To track the eco-evolutionary dynamics of bacteriophages in a complex system, we analyzed 174 shotgun metagenomes from Quistad *et al*. (*36*), a long-term mesocosm experiment with compost communities. We briefly summarize the experimental setup below.

**Fig. 1.**
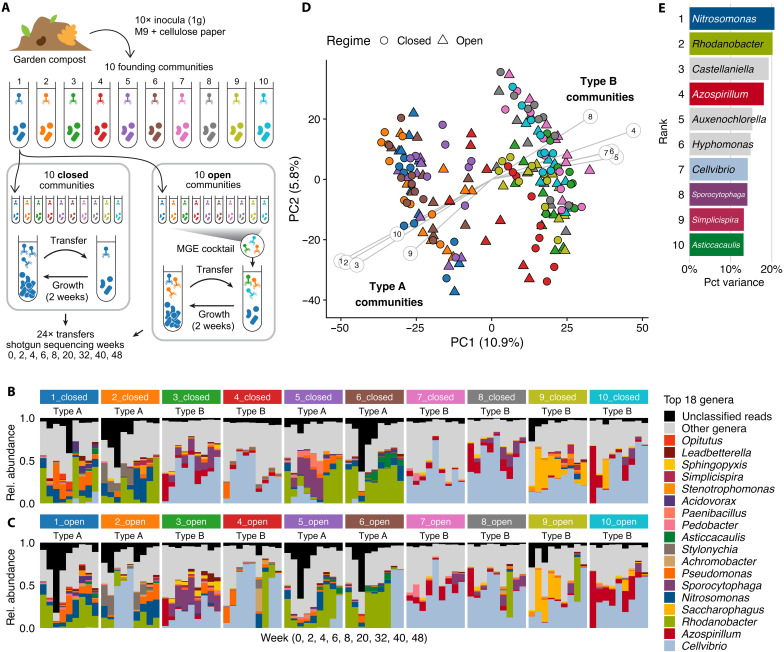
Compost communities cluster in two stable, distinct community types. (**A**) Experimental design of Quistad *et al.* (*36*). Ten founding communities were established from garden compost and propagated on paper cellulose paper by serial transfer (2 weeks) for 48 weeks. In the closed regime, communities were propagated in isolation. In the open regime, a MGE cocktail was added at every transfer, consisting of pooled, 0.2-μm filtered material from all open communities (Materials and Methods). (**B**) Relative abundance (fraction of mapped metagenome reads) of the 18 most abundant genera in closed mesocosms (annotated with RAT). Unclassified reads (black) often represent viral reads (see Fig. 2). (**C**) Same as (B), for open mesocosms. (**D**) PCA of centered log-ratio transformed genus-level relative abundances reveals two distinct community types along PC1: type A, *Rhodanobacter*-dominated and type B, *Cellvibrio*-dominated. Community type of mesocosms is stable over time and consistent across paired closed and open mesocosms, suggesting a strong founder effect. Gray lines indicate the top 10 genera contributing most to PC1 and PC2, numbered as in (E). For separate PCAs of closed and open regimes, see fig. S8. (**E**) Proportion of variance in PC1 and PC2 explained by the top 10 contributing genera. Numbers correspond to (D) and colors match (B) and (C).

Ten independent founding communities were established by sampling 1 g of garden compost from different locations on a compost heap. Each community was incubated in mesocosms containing 20-ml nitrogen-limited minimal M9 medium supplemented with paper cellulose (4 cm^2^) as the sole carbon source (Fig. 1A). Following 2 weeks of incubation, 1 ml of slurry was transferred to 19-ml fresh medium and cellulose paper and then incubated for another 2 weeks. After this acclimatization period (week 0) two transfer regimes were created: a closed regime where each mesocosm was propagated independently and an open regime where bacteriophages and other mobile genetic elements (MGEs) (*37*) were exchanged among mesocosms using a size-filtered MGE cocktail. This setup created paired closed and open mesocosms for each of the 10 founding communities, with the open regime repeatedly exposing MGEs to new hosts and community contexts, potentially affecting their eco-evolutionary dynamics. In the closed regime, every 2 weeks, 1 ml of cellulosic slurry was transferred to 19-ml fresh medium with cellulose paper. In the open regime, transfers were conducted identically, except that 1 ml of MGE cocktail was added. This cocktail was created at each transfer by mixing 1 ml of homogenized community from all 10 open mesocosms, followed by centrifugation and passage through a 0.2-μm filter. Shotgun sequencing was performed at weeks 0, 2, 4, 6, 8, 20, 32, 40, and 48 for both regimes, with additional sequencing of the MGE cocktail at weeks 2, 4, 6, and 8. This enabled us to track viral migration via the MGE cocktail and to compare cellular and viral dynamics in closed mesocosms with their paired open counterparts.

We began by assembling the metagenomes and characterizing community composition, to provide context for viral eco-evolutionary dynamics. Taxonomic annotation of contigs and bins was performed using Contig Annotation Tool (CAT) (*38*), and taxon relative abundances were calculated with Read Annotation Tool (RAT) (*39*) (Fig. 1B; median 96.1% of sample reads mapped; figs. S1 and S2 and Materials and Methods). Consistent with previous studies (*32*, *36*), this revealed diverse communities with mesocosms containing on average 150.3 ± 36.9 genera (means ± SD; mean 58.2% of sample reads annotated at genus rank; table S1 and fig. S1). Principal components analysis (PCA) of genus-rank community composition revealed two distinct community types separated along the first component (Fig. 1, B and D). Mesocosms 1_closed, 2_closed, 5_closed, and 6_closed were dominated by genera including *Rhodanobacter* and *Nitrosomonas* (hereafter community type A), whereas the remaining six closed mesocosms were enriched in *Cellvibrio* and *Azospirillum* (community type B; figs. S4 and S5). These community types were also apparent at higher taxonomic ranks and remained stable over 48 weeks, with dynamics primarily along the second PC (figs. S6 and S7).

The two distinct community types were consistent across all paired closed and open mesocosms, suggesting a strong founder effect (Fig. 1, C and D). This may reflect local subcommunities already present in different regions of the compost heap at the time of sampling or, alternatively, two distinct ecological attractors that emerged during the acclimatization period preceding the first sequenced time point. Stochastic diversity loss during acclimatization of natural communities to laboratory conditions can result in divergent communities and multistability (*31*–*33*). Biopolymer degrading communities are structured by hierarchical cross-feeding and substrate preferences, with different degraders producing distinct metabolite profiles that influence downstream community assembly of specialized byproduct consumers (*30*). We hypothesize that community type A and B are structured around different cellulose degraders, as *Cellvibrio* and *Rhodanobacter* are both associated with cellulose degradation and metagenome-assembled genomes (MAGs) of these genera assembled from this dataset contain cellulases (*37*, *40*, *41*). However, because cellulases are a highly heterogeneous enzyme group and their predicted genomic presence does not always indicate cellulolytic activity (*42*), this remains to be experimentally validated.

### Massive, parallel outbreaks of a previously uncharacterized *Schitovirus* in type A communities

**Fig. 2.**
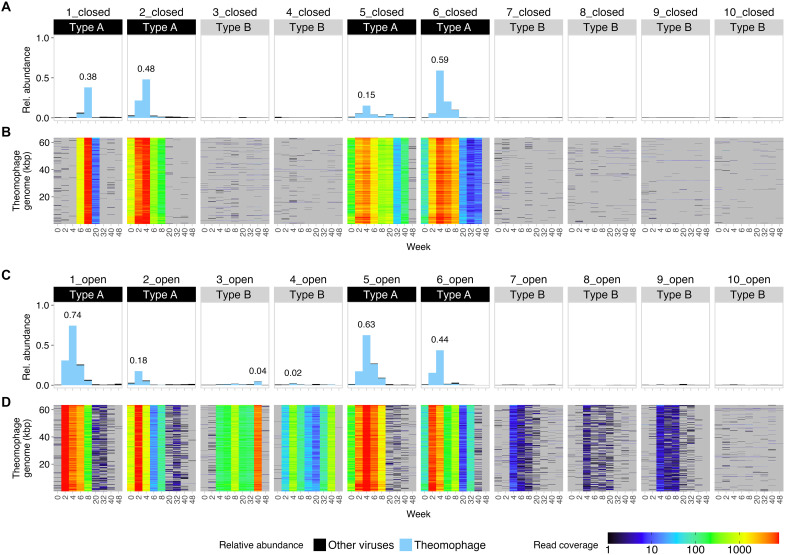
Massive outbreaks of a Theomophage, a previously uncharacterized Schitoviridae bacteriophage, are linked to community background. (**A**) Relative abundance of all predicted viral sequences (black) and Theomophage (blue), a highly abundant, previously undescribed bacteriophage belonging to the family Schitoviridae. Theomophage accounted for up to 59.1 and 74.3% of total community reads in closed (A) and open (C) samples, respectively. Read fractions >1% are labeled. (**B**) Genome coverage profiles show the percentage of sample nucleotides mapped to each position of the Theomophage genome. Theomophage was natively present in all closed type A communities (1_closed, 2_closed, 5_closed, and 6_closed) but absent or very rare in all closed type B mesocosms. Gray indicates no mapped reads. (**C**) same as (A) for 10 open mesocosms. (**D**) Same as (B) but for open mesocosms. Theomophage was detected in five of the six type B mesocosms in the open regime but was absent from their paired closed mesocosms, indicating successful invasion in the open regime.

To investigate the viral dynamics in the compost mesocosms, we screened the assembled metagenomes using WhatThePhage (*43*), a pipeline that integrates 12 virus identification tools (Materials and Methods). This analysis identified 140,378 predicted viral contigs, including 33 complete genomes, 131 high-quality (>90% complete), and 234 medium-quality genomes [50 to 90% complete, estimated with CheckV (*44*)]. Among these, one 63,535–base pair (bp) viral contig (estimated 100% complete; Materials and Methods) stood out due to its high abundance. Further analysis identified this contig as a representative of a new subfamily within the bacteriophage family Schitoviridae, which we named Theomophage after its place of origin, Place Théodore-Monod in Paris, France (see the “Theomophage represents a new subfamily within Schitoviridae” section). Between weeks 4 and 8 of the experiment, massive outbreaks of Theomophage occurred in all closed and open type A mesocosms, with no outbreak detected in any type B mesocosm (Fig. 2, A and C). These outbreaks reached abundances of up to 74.3% of total community reads before declining (Fig. 2, A and C). Notably, the outbreaks in open mesocosms were often larger or sometimes earlier than in the paired closed mesocosms (Fig. 2, A and C; mesocosms 1_open, 2_open, and 5_open), suggesting that Theomophage migration between open mesocosms accelerated and intensified outbreaks. Note that unclassified reads, including Theomophage reads, were not included in the PCA that delineated type A and type B communities, so Theomophage outbreaks did not drive the separation between community types.

To determine whether Theomophage was present in the six type B communities where no outbreaks were detected, we inspected its genome coverage across these samples (Fig. 2B and Materials and Methods). This revealed that Theomophage was absent or very rare in closed type B communities (means ± SD, genome breadth of coverage of ≥1 read: 2.44 ± 2.18%; fig. S9 and table S2). In contrast, Theomophage was detected in five of the six open type B communities, where it reached up to 4.5% of total community reads and persisted for as long as 38 weeks (Fig. 2D). Furthermore, complete Theomophage genome was assembled from and detected in all sequenced MGE cocktail samples (fig. S10). Together, this indicates that Theomophage was originally present in type A founding communities and successfully colonized almost all type B mesocosms via migration in the open regime.

Despite successful colonization, Theomophage abundances were an order of magnitude lower in type B communities than in its native type A context, indicating a strong effect of community type on ecological success. This observed lower abundance could be due to several factors. Host availability may have been lower in type B mesocosms or hosts may have exhibited higher levels of resistance. It is also possible that the original host of Theomophage was absent in type B communities, forcing it to infect a new, sub-optimal host. Although unlikely, due to the compositional nature of metagenomics, higher abundances of other taxa in type B mesocosms could have reduced the relative abundance estimates of Theomophage despite similar absolute abundances.

### Theomophage represents a new subfamily within Schitoviridae

**Fig. 3.**
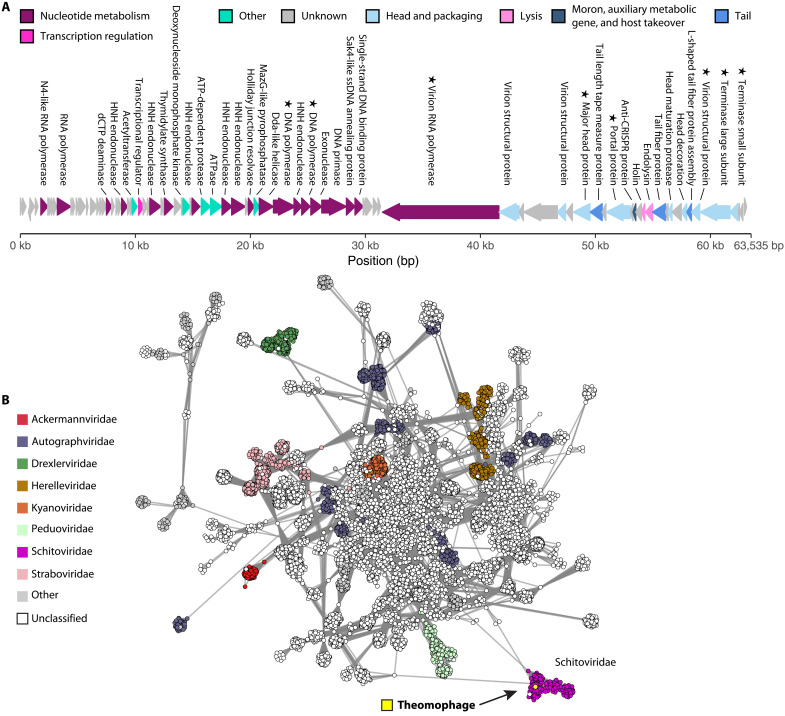
Genomic architecture and gene-sharing network of Theomophage, a previously uncharacterized Schitoviridae bacteriophage. (**A**) Theomophage genome (63,535 bp), encoding 88 genes and one tRNA, including all 7 Schitoviridae hallmark genes (indicated with stars). Gene functions for 40 genes were predicted with Pharokka (*99*) and Phold (*100*) and hallmark genes identified with HMM searches. (**B**) vConTACT2 (*45*) gene-sharing network of Theomophage and 26,302 bacterial and archaeal viruses from the INPHARED database (*113*). Circles represent viral genomes, with edges indicating degree of connectivity based on shared protein clusters. Theomophage groups with bacteriophages of the family Schitoviridae (purple) but is only distantly related to known Schitoviridae sequences, suggesting that it represents a new subfamily (figs. S15 and S16). Only the subnetwork containing Theomophage is shown. ATP, adenosine 5′-triphosphate; dCTP, 2′-deoxycytidine 5′-triphosphate; ATPase, adenosine triphosphatase.

To determine the taxonomic affiliation of Theomophage, we constructed a gene sharing network using vContTACT2 (*45*) and the INPHARED viral reference set containing 26,302 bacterial and archaeal viral genomes (Fig. 3B and Materials and Methods). Theomophage grouped with Schitoviridae (previously N4-like phages), a globally distributed family of double-stranded DNA bacteriophages with podovirus-like morphology (*46*). As is typical for Schitoviridae, the genome consists of two large blocks on opposing strands (Fig. 3A and fig. S11). It codes for 88 predicted genes and a single transfer RNA (tRNA), including all seven proposed Schitoviridae hallmark genes (table S3 and Materials and Methods) (*47*). Individual gene phylogenies of terminase large subunit (TerL), major capsid protein (MCP), and portal protein also place Theomophage among the Schitoviridae, further supporting this taxonomic assignment (figs. S12 to S14).

To assess the phylogenetic placement of Theomophage within Schitoviridae, we followed the current standards of International Committee on Taxonomy of Viruses (ICTV) for Schitoviridae species, genera, and subfamily demarcation, defined as ≥95, ≥70, and ≥40% intergenomic similarity, respectively (*46*). We calculated the pairwise intergenomic similarities of Theomophage and 573 high-quality (>90% complete) Schitoviridae genomes and MAGs from three recent studies (*46*–*48*) using VIRIDIC (*49*). The closest relative, a 72-kbp MAG coassembled from human fecal samples (IMGVR_UViG_3300045988_071216), shared only 8.98% intergenomic similarity with Theomophage (fig. S15 and Materials and Methods), well below the 40% ICTV threshold for subfamilies, or the more relaxed ≥20% threshold proposed by Zheng *et al.* (*47*). Gene-sharing analysis further supported the high divergence of Theomophage to known Schitoviridae sequences. Using vConTACT2, we found that Theomophage did not group with any of the 573 Schitoviridae genomes in vConTACT2 subclusters, which align with genus-level taxonomy as defined by the ICTV (fig. S16 and Materials and Methods) (*45*). Together these results (intergenomic similarity, hallmark gene presence, and genome architecture) indicate that Theomophage represents a novel subfamily in Schitoviridae.

To predict the Theomophage lifestyle, we used BACPHLIP (*50*), which classified it as lytic (score of 0.9625), a lifestyle common in Schitoviridae (*47*). Further evidence for this lifestyle prediction comes from the observation that none of the 54 independently assembled Theomophage viral MAGs (vMAGs), all with 100% estimated completeness, contained bacterial flanking regions indicative of integration into a host genome (see Materials and Methods).

Cellulase activity has been reported for Schitovirus vB_EamP-S6 virions (*51*), prompting us to investigate whether the high abundance of Theomophage in the cellulose-degrading communities might be related to cellulolytic capability. We queried the Theomophage genome using TBLASTN with vB_EamP-S6 cellulase genes Gp94, Gp95, and Gp96. No significant matches were found (table S11), suggesting that the ecological success of Theomophage is not linked to cellulose degradation.

### Community composition remains stable despite massive Theomophage outbreaks

Viral predation can regulate microbial populations and drive major shifts in community composition and function (*34**,* *52*). For example, viral outbreaks following induced coccolithophore blooms can shift the balance between eukaryotic and bacterial organic matter recyclers (*52*), and bacteriophage outbreaks are implicated in restoring gut microbial diversity following antibiotic treatment (*53*). We hypothesized that the massive Theomophage outbreaks in type A mesocosms would similarly impact community composition. However, Bray-Curtis dissimilarity between samples taken before and after Theomophage outbreaks was no greater than shifts over similar intervals in mesocosms without outbreaks (fig. S17), suggesting that the communities were resilient to the Theomophage outbreaks. This observation is consistent with the emerging view of a potentially limited impact of viral predation on microbial community structure (*2*).

### Candidate hosts of Theomophage

To identify potential hosts, we combined three complementary approaches: CRISPR spacer matching, host prediction using iPHoP, and abundance co-occurrence analysis. Screening all mesocosm and cocktail metagenomes for CRISPR spacers with Spacerextractor (*54*) recovered 298,447 spacers, none of which matched the Theomophage genome (≤3 mismatches), providing no CRISPR-based evidence of host interaction within the compost dataset (Materials and Methods). A broader search against SpacerDB (*55*) identified one spacer with a single mismatch linked to a *Moraxella* genome from a wastewater sample and six spacers with two to three mismatches from *Nitrosomonas* MAGs (table S10). The high abundance of Theomophage in compost mesocosms suggests infection of a dominant community member. As *Moraxella* was detected only at very low abundance and showed no association with Theomophage presence across mesocosms (maximum of 182 reads; 0.0005% per sample; fig. S18C), we consider it an unlikely host. In contrast, *Nitrosomonas* was abundant in type A communities where Theomophage outbreaks occurred and was also present in two open type B mesocosms where Theomophage invaded and reached high abundance (3_open and 4_open; fig. S18A). Although spacers with two to three mismatches provide weak evidence of a phage-host relationship, the co-occurrence of *Nitrosomonas* and Theomophage across mesocosms supports a potential phage-host relationship.

Because only ~40% of bacteria encode CRISPR-Cas defense systems (*56*), we complemented the CRISPR-based analysis with iPHoP, a machine-learning framework integrating multiple phage-host prediction tools (*57**–**61*). iPHoP returned JACPRH01, a family within the Nitrososphaerales order of soil ammonia-oxidizing archaea (*62*) as the highest-confidence prediction (iPHoP score of 85.80). Similar to *Moraxella,* JACPRH01 was detected at very low abundance in compost mesocosms and showed no association with Theomophage (fig. S18B). If JACPRH01 is the true host, then it would suggest an unexpected host range expansion of the Schitoviridae family to the archaeal domain [all known Schitoviridae phages infect *Proteobacteria* (*46*)]. Given the limited and indirect nature of the evidence for potential hosts (*63*), we tentatively propose *Nitrosomonas* as a host candidate, although further validation (e.g., isolation or proximity ligation-based Hi-C) is required.

### Migration drives Theomophage evolution by exposure to new community contexts

**Fig. 4.**
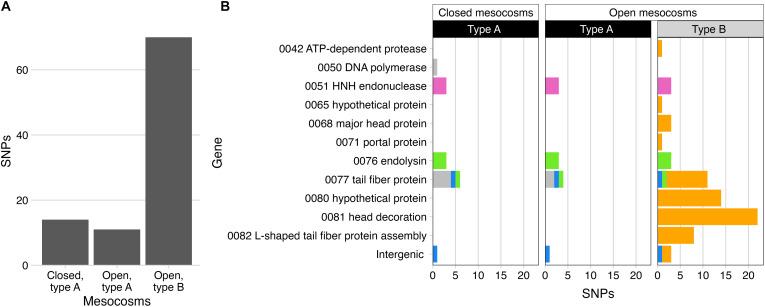
Increased number of SNPs following migration to a new community context. (**A**) Total SNPs detected in Theomophage populations in type A and type B communities under closed and open regimes. Most SNPs (80.3%) were found exclusively in the type B communities, which Theomophage colonized in the open regime. (**B**) Genomic positions of the SNPs in (A), with colors assigned based on similarity of allele trajectories (Materials and Methods; Fig. 5, A, C, and E; and Table 1).

The strong association of Theomophage with type A communities, together with its successful colonization of type B mesocosms, enabled us to investigate how migration and community context shape bacteriophage evolution. We compared three conditions: (i) closed type A mesocosms (no viral migration and native context), (ii) open type A mesocosms (viral migration and native context), and (iii) open type B mesocosms (viral migration and new context). We hypothesized that these conditions would impose distinct selection pressures and result in different evolutionary signatures. In particular, we predicted accelerated molecular evolution in type B communities due to Theomophage’s lower relative abundance in this new community context, consistent with local maladaptation or a possible host switch. To test this, we identified single-nucleotide polymorphisms (SNPs) in Theomophage populations by mapping sample reads to reference assemblies (Materials and Methods). In total, we identified 76 SNPs with frequencies >10% in Theomophage populations. Open type B mesocosms contained the most SNPs (*n* = 70), of which 80.3% (*n* = 61) were unique to type B communities (Fig. 4, A and B, and fig. S19A). This pattern supports the idea that exposure to new community contexts accelerates bacteriophage evolution and diversification.

### Theomophage evolution is constrained in closed, native communities

**Fig. 5.**
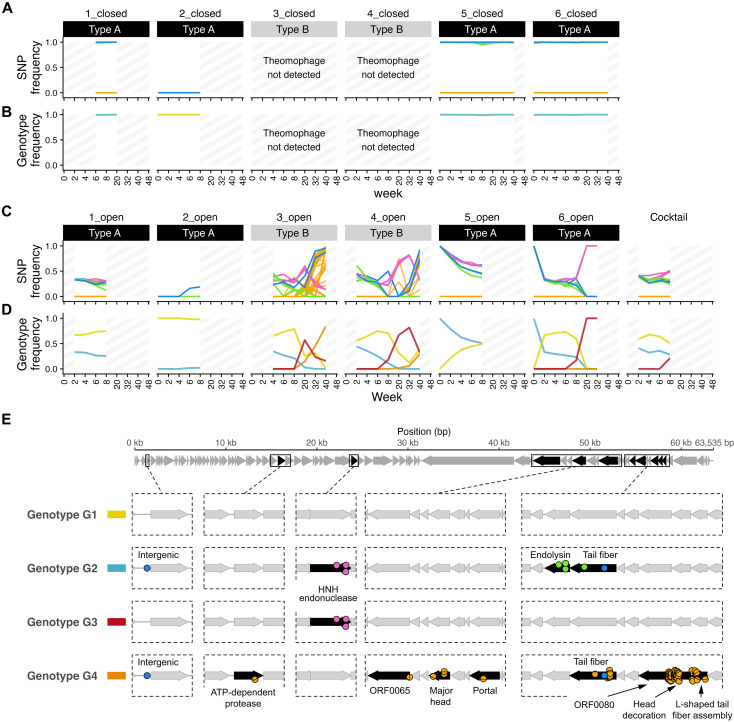
Molecular evolution and migration of Theomophage in closed and open mesocosms. (**A**) Allele-frequency trajectories in closed mesocosms of SNPs that differed from the reference genotype G1. Theomophage populations in type A mesocosms were initially monomorphic: 2_closed contained genotype G1 and mesocosms 1_closed, 5_closed, and 6_closed contained genotype G2, which differed from G1 by nine SNPs [allele frequencies near 1.0, see (E)]. No new SNPs emerged that fixed in closed mesocosms during the 40 weeks over which Theomophage was detected. Shading indicates samples with insufficient coverage for variant calling or in which Theomophage was not detected. (**B**) Inferred genotype dynamics from (A); each mesocosm contained either G1 or G2. Colors correspond to (E). (**C**) Same as (A), but for open mesocosms and MGE cocktail. Both genotypes spread via the MGE cocktail, cross-invading type A mesocosms and previously Theomophage-free type B mesocosms. Decoupling of previously linked SNP trajectories in 3_open and 6_open at weeks 6 to 8 indicates two independent recombination events between G1 and G2, giving rise to new genotypes G3 and G4. G4 additionally acquired 61 additional SNPs (orange). (**D**) Genotype dynamics in open mesocosms. Samples from 7_open, 8_open, and 9_open not shown due to insufficient read coverage for variant calling, although Theomophage was sparsely detected there (Fig. 2). (**E**) Genomic positions of SNPs distinguishing genotypes G1 to G4. Most (46 of 61) new SNPs in G4 occurred in ORF0080, head decoration protein and L-shaped tail fiber assembly protein (Table 1 and table S5). SNPs are vertically jittered for clarity. SNP colors match (A) and (C), Fig. 4B, and Table 1 and are assigned on the basis of similarity of allele-frequency trajectories (Materials and Methods).

Next, to explore in more detail how these SNPs emerged and spread, we reconstructed Theomophage genotypes and tracked them over time and across mesocosms. Reconstructing genotypes from short-read data is challenging because SNPs typically lie on different reads, making it hard to resolve linkage. Here, we leveraged the observation that many samples were monomorphic at all variant positions, indicating clonal populations (i.e., all allele frequencies near 0 or 1; Materials and Methods). Using this approach, we identified two genetically distinct Theomophage genotypes in the founding communities. Mesocosm 2 contained a genotype identical to the assembled vMAG used for variant calling (hereafter genotype G1), while mesocosms 1, 5, and 6 harbored a second genotype (G2), which differed from G1 by nine SNPs located in genes associated with host interaction and replication, including the tail fiber protein, HNH endonuclease, endolysin, and one intergenic region (Fig. 5, A, B, and E, and Table 1). Each founding mesocosm contained a single genotype, with minimal within-population diversity.

**Table 1.** Genomic variants defining four Theomophage genotypes. For each SNP, the position, effect, associated open reading frame (ORF), predicted gene function, and presence in inferred genotypes are shown. Nine SNPs differentiate genotype G2 from G1. Details for SNPs arising in G4 are provided in table S5. Colors match Figs. 4B and 5.

Variant	Effect	ORF	Predicted function	G1	G2	G3	G4	Color in Fig. 4/5
1276 T → G	Intergenic	–	–	–	√	–	√	Blue
24224 T → C	Ile → Thr	0051	HNH endonuclease	–	√	√	–	Pink
24375 G → A	Synonymous	0051	HNH endonuclease	–	√	√	–	Pink
24378 T → C	Synonymous	0051	HNH endonuclease	–	√	√	–	Pink
54689 T → C	Glu → Gly	0076	Endolysin	–	√	–	–	Green
54853 T → C	Synonymous	0076	Endolysin	–	√	–	–	Green
54856 C → T	Synonymous	0076	Endolysin	–	√	–	–	Green
55320 A → C	Val → Gly	0077	Tail fiber protein	–	√	–	–	Green
55814 C → T	Synonymous	0077	Tail fiber protein	–	√	–	√	Blue
16296 T → A	Leu → Ile	0042	ATP-dependent protease	–	–	–	√	Yellow
22232 A → G	Synonymous	0050	DNA polymerase	–	–	–	√	Yellow
46679 C → T	Gly → Ser	0065	Hypothetical protein	–	–	–	√	Yellow
3 SNPs	See table S5	0068	Major head protein	–	–	–	√	Yellow
51895 G → T	Leu → Ile	0071	Portal protein	–	–	–	√	Yellow
14 SNPs	See table S5	0077	Tail fiber protein	–	–	–	√	Yellow
14 SNPs	See table S5	0080	Hypothetical protein	–	–	–	√	Yellow
2 SNPs	Intergenic	–	–	–	–	–	√	Yellow
22 SNPs	See table S5	0081	Head decoration	–	–	–	√	Yellow
8 SNPs	See table S5	0082	L-shaped tail fiber protein assembly	–	–	–	√	Yellow

With the identity and initial distribution of genotypes established, we next tracked how these genotypes changed over time within each mesocosm. In closed mesocosms, no new SNPs appeared in either genotype G1 or G2 over the 40 weeks in which Theomophage was detected, although genotype G1 contained limited preexisting microdiversity in mesocosms 5_closed and 6_closed (Fig. 5A and fig. S18). The absence of novel mutations is striking, given the massive phage outbreaks and the long duration over which Theomophage was observed, which imply strong viral predation and opportunities for antagonistic coevolution with its host. In experimental evolution studies in simplified phage-host systems (*4*, *26*) and low-diversity dairy starter cultures (*64*), phage evolution is typically rapid and driven by antagonistic coevolution. In contrast, we observed prolonged genetic stability under ecologically complex conditions. Consistent with previous observations from natural environments (*17*–*20*), our results indicate that phage evolution in complex microbial communities may proceed only slowly, with long-term, stable coexistence between phage and host.

### Migration accelerates evolution via recombination between preexisting Theomophage genotypes

In contrast to the genetic stability observed in closed mesocosms, Theomophage populations in open mesocosms exhibited rapid and complex evolutionary dynamics, driven by genotype migration, recombination, and the acquisition of new SNPs following colonization of the type B mesocosms. While closed mesocosms only contained either genotype G1 or G2, in open mesocosms, both genotypes were often detected immediately following the first serial transfer, indicating rapid dissemination between mesocosms (Fig. 5, C and D). For example, mesocosm 5_open initially contained only genotype G2, but by week 2, genotype G1 had invaded and rose to ~50% frequency by week 8, as indicated by the coupled frequency decline of the nine SNPs that characterized G2. Similar cross-invasions were observed in all other open mesocosms with sufficient coverage for variant calling, including colonization of the novel type B mesocosms by both Theomophage genotypes. Both migrating genotypes were also detected in the MGE cocktail used for transfers between mesocosms, providing further evidence that these patterns resulted from viral migration rather than parallel evolution (Fig. 5, C and D).

In all open mesocosms where G1 and G2 coexisted beyond week 8, the frequency trajectories of their characteristic alleles became decoupled, indicating recombination between the genotypes. For example, in 6_open, genotype G1 and G2 coexisted for 8 weeks, after which a recombinant genotype (G3) emerged, characterized by three G1-derived alleles in HNH endonuclease and six G2-derived alleles (Fig. 5, C and D, and Table 1). Similarly, in 3_open, a second recombinant genotype (G4) arose that combined alleles from both founder genotypes. Both recombinants replaced their parental genotypes and spread to other mesocosms via continued migration. G3, originating in 6_open, was detected in 3_open and 4_open, while G4 spread from 3_open to 4_open and eventually to 6_open by week 32, as revealed by a more sensitive SNP analysis (fig. S20).

### Rapid SNP accumulation following migration to new community type

At week 8, 61 new SNPs appeared in 3_open that were not detected in any founding or closed communities (Fig. 5, C and D). The close similarity in frequency trajectories between these novel SNPs and the defining alleles of G4 indicates that they were located on the same genome. These mutations occurred in several predicted structural proteins, including major capsid and tail fiber protein, but were mostly (46 of 61 SNPs) clustered in a region spanning three consecutive genes: a gene of unknown function (ORF0080), head decoration protein (ORF0081), and L-shaped tail fiber assembly protein (ORF0082; Fig. 5E and table S5). Tail fibers and head decoration proteins are involved in host receptor binding (*65*, *66*) and are often under selection during a host switch (*67*, *68*). Therefore, the concentrated accumulation of mutations in these host-recognition genes is consistent with adaptation to a new host following Theomophage migration into type B communities. This interpretation is supported by the timing of their appearance following migration to type B mesocosm 3_open, the subsequent increase in Theomophage abundance after fixation of these SNPs (week 40; Fig. 2C) and the later spread of this genotype to 4_open and 6_open, suggesting a selective advantage in these communities.

To investigate the mechanistic origin of these mutations, we considered several known mechanisms of viral evolution. We first tested whether they arose de novo via diversity-generating retroelements (DGRs)—error-prone reverse transcriptase systems that target specific genomic loci, typically structural genes, to promote rapid adaptation to new hosts (*69*). Screening the Theomophage genome with MyDGR (*70*) revealed no DGRs or reverse transcriptase genes, making this mechanism unlikely. Another major driver of viral evolution is recombination (*21*). A BLASTn (*71*) query of all assembled contigs with the Theomophage region spanning the three mutated genes yielded no significant matches, providing no evidence for recombination with assembled sequences. However, previous work has shown that in natural ecosystems, rare viral genotypes (e.g., below the metagenomics detection threshold) can act as viral seed banks and harbor genetic diversity that can become adaptive when selection pressures change (*72*). These members of the rare virosphere could therefore act as recombination partners. We hypothesize that following migration to type B mesocosm 3_open, Theomophage recombined with a rare viral sequence that was not captured by our assemblies, giving rise to genotype G4. The tight genomic clustering of novel mutations in G4 further supports recombination, rather than independent point mutations, as the likely source of these variants.

## DISCUSSION

Factors that shape the eco-evolutionary dynamics of bacteriophages in complex natural communities—including microdiversity, community context, and migration between local subcommunities—are often absent from laboratory systems that trade genomic and spatiotemporal complexity for experimental tractability. These simplified systems have provided foundational insights into bacteriophage ecology and evolution, but their outcomes do not always generalize to natural communities (*8*–*11*, *23*, *24*). To address this gap, we analyzed longitudinal metagenomic data from diverse, compost-derived microbial communities grown under controlled conditions to determine how community context and viral migration shape viral evolution and population dynamics.

We captured evidence of massive, parallel outbreaks of Theomophage, a previously undescribed bacteriophage, across different independent compost communities, where it reached unprecedented abundances of up to 74.3% of metagenome reads. This dominance by a single virus is rare: Viral reads typically constitute ~5% of metagenomic sequences (*73*), although higher values have been reported. For example, crAssphage can reach 22% in human gut metagenomes (*74*) and a *Streptococcus* phage comprised ~20% of reads in cheese starter cultures composed of two bacteria and three associated phages (*75*). Our findings far exceed these cases, showing that a single 63-kb phage can dominate even highly diverse microbial communities in terms of genomic information.

Viral abundance is a key parameter in microbial ecology but remains challenging to measure in complex microbial communities (*76*, *77*). Metagenomics allows quantification of both free and intracellular viruses and enables genome-level characterization, making it particularly suited to identifying specific viral taxa in complex communities (*78*). Is it biologically plausible for 74% of nucleotides in a community metagenome to derive from a single bacteriophage, given that phage genomes are far smaller than those of their hosts? A first-order approximation based on genome sizes indicates that these values are feasible. Assuming a typical bacterial genome size of ~4 Mb, Theomophage (63 kb) would need to reach a viral-to-bacterial genome ratio of ~174 to account for 74% of metagenomic reads. Metagenomics-based virus-to-microbe ratios above 100 are high but have been reported in natural communities (*78*). However, in our system, this ratio is driven by a single phage, and given the narrow host range of bacteriophages and high community diversity of the compost samples, only a fraction of the bacterial population would have served as hosts for Theomophage. If fewer bacteria served as hosts, then the required viral burst size would increase proportionally. For example, if the Theomophage host constituted ~10% of the bacterial community, then the required burst size would increase to ~1740 virions per host cell. Although high, burst sizes in this range can occur under certain conditions, including those found in our system (*79*, *80*).

One such factor is lysis inhibition (LIN). LIN is a viral strategy in which infected cells delay lysis upon superinfection, allowing extended intracellular viral replication and substantially increasing viral yield under host-limited conditions. The prototypical Schitovirus N4 undergoes LIN, with reported burst sizes of 3000 (*79*). Serial-transfer protocols such as the one used here create favorable circumstances for LIN (*80*), as virions accumulate during the 2-week growth period while the host population depletes, increasing the likelihood of superinfection events. Free virions are also carried over between transfers, enabling phage outbreaks to grow across growth cycles, and are more stable than extracellular bacterial DNA released during lysis, which is rapidly degraded and recycled. These features increase the representation of viral genomes in metagenomic sequencing at the end of the growth cycle.

Beyond biological factors, metagenome-derived viral abundance estimates may also be influenced by technical biases introduced during DNA extraction, library preparation, and sequencing (*76*, *77*, *81*). Rolling circle amplification, which can strongly distort viral abundance estimates (*82*), was not used here. The Nextera XT library preparation kit used in this study may underrepresent regions with low guanine-cytosine (GC) content (*83*), but given that Theomophage has a lower GC content (34%) than the average of the assembled metagenomes, this bias would underestimate rather than inflate its abundance. Our estimates of Theomophage abundance may therefore be conservative. Together, these biological mechanisms and technical considerations indicate that the extreme abundances of Theomophage observed in our metagenomes fall within the bounds of biological plausibility.

The extraordinary abundance of Theomophage highlights the importance of accounting for viral sequences when analyzing metagenomes. State-of-the-art taxonomic profilers used in metagenome analysis struggle to identify viral sequences (*84*), requiring the use of specialized viral identification tools (*85*, *86*). Comprehensive viral profiling remains limited to well-characterized biomes (*73*), and the terrestrial virosphere in particular remains underexplored (*87*), as illustrated by our discovery of a previously uncharacterized subfamily within Schitoviridae. This means that some of the most abundant members of microbial communities may be missed in metagenome profiling, underscoring the need for viral-aware metagenomics approaches to accurately characterize microbial ecosystems.

Viruses are commonly assumed to be rapidly evolving entities due to their high mutation rates and parasitic lifestyle, which impose strong selection that drives ongoing coevolution with their hosts. This view is supported by simplified laboratory systems composed of single phage-host pairs, which typically exhibit rapid, antagonistic coevolution (*4*, *5*, *16*). In contrast, we observed no detectable Theomophage evolution over 40 weeks of propagation in closed mesocosms despite massive outbreaks. These extreme fluctuations in viral abundance imply substantial host mortality, which should in principle generate strong selection for host resistance and corresponding viral counter adaptations. Yet, we did not observe any molecular change in Theomophage populations, suggesting that community context can constrain viral evolution and sustain extended periods of evolutionary stasis.

Migration fundamentally altered this evolutionary landscape. Dispersal among communities in the open regime promoted rapid recombination between preexisting Theomophage genotypes, while migration into new community types facilitated the accumulation of many novel mutations concentrated in host recognition genes, likely via recombination with an undetected viral sequence. These novel genotypes subsequently spread across mesocosms and replaced ancestral lineages. Thus, in the metacommunity context, migration both homogenized genetic variation among local communities and acted as an evolutionary catalyst by exposing Theomophage to new community types and potential hosts.

Together, our findings support a model in which phage-host coevolution in complex ecosystems proceeds through extended periods of evolutionary stasis punctuated by bursts of evolution triggered by dispersal. Following migration, phage-host communities become locally adapted via rapid evolution and reach eco-evolutionary equilibria that persist until the community structure is disrupted. This model is consistent with observations from natural ecosystems in which viral lineages remain genetically stable for years (*19*, *20*) yet exhibit strong local adaptation (*26*, *88*) and long-term coexistence of susceptible and resistant hosts without rapid turnover (*17*, *18*). It further suggests that environmental processes that disturb local community structure, such as seasonal rainfall in soils (*25*, *89*) or cycles of dispersal and community assembly on marine organic particles (*90*), may act as catalysts of bacteriophage evolution in nature.

Last, we highlight potential limitations of our study. First, the coarse temporal resolution in the later stages of the experiment may have caused us to miss additional Theomophage or other phage outbreaks. The more frequent sequencing earlier in the experiment showed that outbreaks took 6 to 8 weeks, suggesting that similar events may have occurred undetected later on. This also limited our ability to assess the impact of Theomophage outbreaks on community composition. Second, the identification of the Theomophage host would provide valuable context for interpreting the evolutionary dynamics of Theomophage. A confirmed host could clarify whether the observed genotype-level diversity in native type A communities reflects local adaptation and whether the increased evolutionary rates following migration to new community types are mirrored in host evolution, or a signature of adaptation to a new, suboptimal host (*68*). If *Nitrosomonas* were confirmed as the host, then this could suggest a host switch following migration to mesocosms 7_open, 8_open, and 9_open, where *Nitrosomonas* was sparsely detected.

Our analysis revealed the central role of migration as a catalyst of bacteriophage evolution by exposing viral populations to new community contexts and enabling diversification through recombination and novel mutations. Moreover, our results demonstrated that a single experimental system can capture two extremes of bacteriophage evolution: the rapid diversification commonly observed in pairwise phage-host setups (*4*, *5*, *16*) and the prolonged evolutionary stasis recently reported in natural ecosystems (*19*, *20*). We also uncovered large, reproducible bacteriophage outbreaks that were tied to a particular community state, demonstrating how ecological context shapes viral population dynamics. More broadly, our study underscores the promise of laboratory-propagated natural microbial communities for uncovering the mechanisms that govern viral eco-evolutionary dynamics in nature.

## MATERIALS AND METHODS

### Data

We analyzed time series of 2 × 10 paired compost mesocosms from (*36*). A brief description of the experimental setup is provided at the beginning of the results section for clarity, and full experimental details are available in (*36*). Note that the closed and open treatments were previously referred to as “vertical” and “horizontal,” respectively (*36*, *37*).

### Assembly and binning

Read quality control was done with Prinseq v.0.20.4 (*91*) using parameters -derep 14 -lc method dus -c threshold 20. Adapters were trimmed using Flexbar v.3.5.0 (*92*) with --adapter-preset Nextera -ap ON. The high complexity of compost ecosystems makes metagenome assembly difficult, potentially leading to short contigs and a large fraction of unassembled reads (*93*). To improve assembly and increase the chance of recovering low-abundant genomes such as bacteriophages (*87*, *94*), reads from all time points for each mesocosm were pooled and coassembled with metaSPAdes v.3.14.0 (*95*). To preserve sample-level diversity for Theomophage genome annotation and variant calling, all samples (*n* = 170) were also assembled separately. Samples from the viral cocktails were assembled in the same way. Reads were mapped using BWA-mem2 v0.7.17-r1188 (*96*), and coverage was calculated with SAMtools v1.7 (*97*). Contig depths were calculated with jgi_summarize_bam_contig_depths and binned using Metabat2 v.2.12.1 (*98*).

### Taxonomic profiling

Taxonomic classification of assembled scaffolds and bins was performed with CAT v5.3 (*38*) with --index_chunk 1 --block_size 5 --top 11 --I_know_what_Im_doing using CAT National Center for Biotechnology Information database and taxonomy v2021-04-30. Taxon abundances (fraction of total sample reads) were calculated using RAT version September 2021 (*39*) and database v2021-04-30. For dimension reduction, unclassified taxa or taxa that were only classified at other taxonomic ranks were dropped, and relative abundances were centered log-ratio transformed, with zero values adjusted by adding 1 to all read counts. To estimate community richness, the number of detected genera was counted with different abundance cutoffs (table S1). Reads from contigs that could not be annotated at genus level by RAT, e.g., due to conflicting information from open reading frames (ORFs) or incomplete taxonomic annotation of best matches, were either counted as a separate genus or removed. Putative viral sequences were identified with WhatThePhage v0.9.0 (*43*). While counting any contig marked as viral by this panel of virus identification tools likely increased the number of false positives, our subsequent analysis focused on a single bacteriophage sequence identified with high confidence by manual curation. To estimate viral abundances (including Theomophage), we randomly selected a coassembly where Theomophage was completely assembled and used that as a target for read mapping.

### Viral completeness estimation, annotation, lifestyle prediction

Viral contig completeness was estimated using CheckV v1.0.3 with database v1.5 (*44*). Theomophage contig NODE_224 was estimated 100% complete based on the presence of 55-bp direct terminal repeats (DTRs). Complete Theomophage genomes were independently assembled in 54 independent, single time-point assemblies, with a consistent contig length of 63,535 bp (46 of the 54 contigs; fig. S21) and high sequence similarity [99.98 average nucleotide identity (ANI)]. The genomes were circularly permuted and contained DTRs, consistent with complete genome assembly from shotgun reads derived from phage replication concatemers or linear genomes with DTRs (fig. S22).

Theomophage MAG NODE_224_length_63535 assembled from sample 3_open-week_4 was annotated with Pharokka v1.7.5 (*99*) and Phold v0.2.0 (*100*) using default settings. Specifically, coding sequences (CDs) were predicted with PHANOTATE (*101*), tRNAs were predicted with tRNAscan-SE 2.0 (*102*), tmRNAs were predicted with Aragorn (*103*), and CRISPRs were predicted with CRT (*104*). Functional annotation was generated by translating protein amino acid sequences to the 3Di token alphabet with ProstT5 (*105*), and Foldseek (*106*) was then used to search these against the Phold phage protein structures database, which contains PHROGs (*107*), VFDB (*108*), and CARD (*109*) structures predicted with Colabfold (*110*). To search for the seven Schitoviridiae hallmark genes, a protein hidden Markov model (HMM) was constructed for each from protein sequences from (*47*) by aligning them with MAFFT v7.526 (*111*) and hmmbuild (HMMER v3.4, www.hmmer.org). The resulting models were used to search the predicted Theomophage CDs using hmmsearch (HMMER v3.4) with default parameters. Output is available as table S4. Figs. 3A and 5E were drawn in R v4.3.1 with gggenes v0.4.1 (https://github.com/wilkox/gggenes) and ggmagnify v0.4.1 (https://github.com/hughjonesd/ggmagnify). Theomophage lifestyle was predicted with BACPHLIP v.0.9.6 (*50*). Figure S11 was created with Clinker v0.0.31 (*112*) with an identity cutoff of 0.24 based on GenBank files for Theomophage MAG NODE_224_length_63535 and *Escherichia* phage N4 (GenBank accession EF056009.1) created with Pharokka as described above.

### Theomophage taxonomy

The gene sharing network was created by combining predicted proteins of 26,302 bacterial and archaeal viral reference genomes from the INPHARED reference set (1 October 2023) (*113*) with predicted proteins from Theomophage NODE_224_length_63535 using vConTACT2 v.0.9.19 (–rel-mode ‘Diamond’ –pcs-mode MCL –vcs-mode ClusterONE). For fig. S16, a gene sharing network was created for Theomophage and 573 Schitoviridae reference sequences (see below). Networks were visualized with Cytoscape v.3.9.1 (*114*) and Adobe Illustrator 2025.

A reference set of 573 high quality (predicted >90% complete) Schitoviridae sequences was created by downloading sequences described as “high quality” or “complete” in (*46*) and (*47*) from Genbank and IMG/VR v3, complemented by adding sequences annotated as “Schitoviridae” and high quality or “isolate” from IMG/VR v4 (*48*), followed by dereplication at 100% ANI with CD-HIT v.4.8.1 (*115*). For gene sharing networks, protein prediction was performed as described above.

For tailed bacteriophages, three conserved proteins—the Terminase large subunit (terl), major capsid protein (MCP), and portal protein—can be used to infer taxonomic relationships (*116*). We gathered proteins annotated as “terminase large,” “portal,” or “major capsid” or “large capsid” from the INPHARED dataset and added the corresponding protein from Theomophage MAG NODE_224. Genes were aligned with Clustal Omega v.1.2.4 (*117*) using default parameters, and positions with gaps in more than 50% of the sequences were removed with trimAl v.1.4.rev15 (*118*). Gene trees were made using FastTree version 2.1.10 (*119*) with default parameters and plotted with R v.4.3.1 using the ggtrees package (*120*).

Intergenomic similarities (pairwise nucleotide identity normalized for genome lengths) were calculated from MAG NODE_224 and 573 high-quality Schitoviridae sequences using VIRIDIC standalone v1.1 (blastn parameters “-word_size 7 -reward 2 -penalty -3 -gapopen 5 -gapextend -2”) (*49*).

### Host prediction

iPHoP v1.3.3, database Aug_2023_pub_rw (*57*) was run with default parameters and additionally with a lowered confidence cutoff (-m 80). iPHoP predictions and raw output per bioinformatic tool are available as tables S6 and S7.

CRISPR spacers were extracted from mesocosm and cocktail reads and filtered and denoised with Spacerextractor (*54*) v.0.9.5 using default parameters. Resulting spacers were mapped against the four inferred Theomophage genotypes (MAG NODE_224_length_63535_cov_5745_934389, genotypes G2, G3, and G4) using Spacerextractor with default parameters, which resulted in no matches. Spacers from the global SpacerDB database v.2025-05-02 (*55*) were mapped in the same way against the four Theomophage genotypes, only considering spacers that mapped with less than four mismatches across the full spacer length. Results are available as tables S8 to S10.

### Variant calling

To reduce the chance of spurious read mapping, quality control–filtered reads were competitively mapped against all contigs from a randomly selected single-sample assembly containing the complete Theomophage MAG (sample 3_open-week_4 containing NODE_224_length_63535_cov_5745_934389) with BWA-mem2 (*96*). Variants were called for the Theomophage MAG using HaplotypeCaller, GenomicsDBImport, and GenotypeGVCFs from GATK v.4.2.3.0 (*121*) following the joint variant calling pipeline and the best practices for germline short-variant discovery. To exclude sequencing and read mapping artifacts, only alleles that reached a frequency of 10% in any sample and were supported by a minimum of four reads, coming from samples where ≥30% of the genome was covered by ≥10 reads were considered for downstream analysis. This resulted in a total of 76 unique SNPs across all samples. To further exclude read mappings artifacts, read depth coverage profiles were inspected with Integrative Genomics Viewer v.2.13.2 (*122*). Variants located in genomic areas with irregular coverage (e.g., abrupt depth changes) were further evaluated by separately reassembling mapped read pairs for these samples and a second round of variant calling using the resulting contig as reference. All SNPs were confirmed, and no variants were removed.

Variant effects were predicted with SNPEff v.5.0e (*123*) (-noLog -noStats -no-downstream -no-upstream -no-utr) using a custom SNPEff database built with NODE_224_length_63535_cov_5745_934389 as reference, a .gff file generated using Prokka and the Bacterial_and_Plant_Plastid codon table. MAGs were interrogated for DGRs with the webserver MyDGR (*70*).

### Resolving genotypes and estimating genotype abundance

Genotypes G1, G2, and G3 were inferred from samples where allele frequencies for all 76 polymorphic sites were near fixation (defined here as >0.97 or <0.03). Specifically, genotype G1 was inferred from sample 2_closed week 0, in which all 76 sites had frequencies of 0, indicating a clonal population identical to the reference genome (Fig. 5, A, C, and E, and Table 1). Genotype G2 was inferred from samples 1_closed week 6, 5_closed week 0, and 6_closed week 0, which showed near-fixed alternative alleles at nine sites located in the tail fiber protein, HNH endonuclease, endolysin, and an intergenic position (median allele frequency = 1.0, mean = 0.996, minimum of 0.971). Genotype G3 was inferred from samples 6_open weeks 20 and 32, in which three sites in HNH endonuclease (24224 T→C, 24375 G→A, and 24378 T→C) were fixed while all other sites had a frequency of 0 (i.e., matched the reference allele). Genotype G4 was inferred from sample 3_open week 40. Although this sample was not near clonal, it showed several SNPs with allele frequencies exceeding 0.8. By the pigeonhole principle, the simultaneous presence of multiple high-frequency variants implied that these variants co-occurred on the same genome in a substantial fraction of the population. For the other samples in the open regime with intermediate allele frequencies, we assumed the simplest explanatory scenario, namely that these samples represent mixtures of the genotypes inferred from near-clonal samples, rather than additional unobserved genotypes. This interpretation was supported by the highly correlated allele-frequency trajectories of SNPs defining each genotype across samples and time, indicating genetic linkage.

For Fig. 5C, we then assumed the simplest scenario that could explain the evolution of G3 and G4 from G1 and G2 that was compatible with the variant trajectories, namely, two separate recombination events and fixation of 61 new variants in G4. We approximated genotype abundances for weeks 8, 20, 32, and 40 in communities 3_open, 4_open, 6_open, and the cocktail as follows: G4 abundance as the average relative abundance of orange and blue variants; G3 abundance as the average of pink variants; G2 as the average of the blue and green variants; and G1 as 1 minus the abundance of G2, G3, and G4. For other time points, the abundance of G2 was estimated as the average of blue, pink, and green SNPs and G1 as 1 minus the abundance of G2. The four genotypes inferred above accounted for 92.1% of the reported SNPs, and we conclude that they captured most of the relevant dynamics of genetic diversity within the Theomophage population. The remaining six SNPs represent further community-specific microdiversity within genotype G2 that was limited to communities 5_closed, 6_closed, and 5_open (fig. S19; indicated in gray in Fig. 4B). For visual clarity, we gave SNPs with similar trajectories from cluster 1 different colors. Unless noted otherwise, figures and analysis were done in RStudio v.2022.12.0 with R v.4.2.0 using cowplot v1.1.1 (github.com/wilkelab/cowplot), Tidyverse v.1.3.2 (*124*), data.table v1.14.6, and ggbeeswarm v.0.7.1.
