# Supplementary material for: Eco-evolutionary dynamics of massive, parallel bacteriophage outbreaks in compost communities
Source: Sci Adv. 2026 May 29;12(22):eaeb8246. doi: 10.1126/sciadv.aeb8246 (PMC13220884; doi:10.1126/sciadv.aeb8246)
Supplement: Supplementary file 1 — Figs. S1 to S22 Legends for tables S1 to S12 [file sciadv.aeb8246_sm.pdf]

Science Advances
AAAS

Supplementary Materials for

Eco-evolutionary dynamics of massive, parallel bacteriophage outbreaks in compost communities

Jeroen Meijer *et al.*

Corresponding author: Jeroen Meijer, jeroen.meijer@uni-jena.de; Bas E. Dutilh, b.e.dutilh@uni-jena.de

Sci. Adv. **12**, eaeb8246 (2026)
DOI: 10.1126/sciadv.aeb8246

The PDF file includes:

Figs. S1 to S22
Legends for tables S1 to S12

Other Supplementary Material for this manuscript includes the following:

Tables S1 to S12

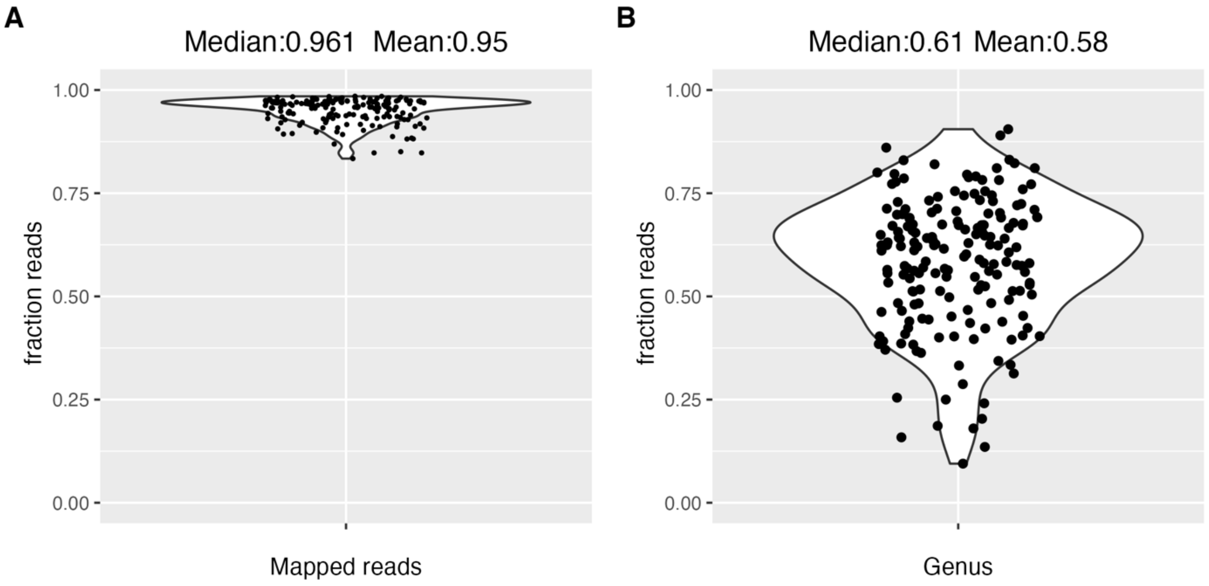

Fig. S1. Fraction of reads mapped to contigs and annotated at genus level.
(**A**) Fraction of sample reads mapped to contigs across 170 samples. (**B**) Fraction of mapped reads assigned to the genus level.

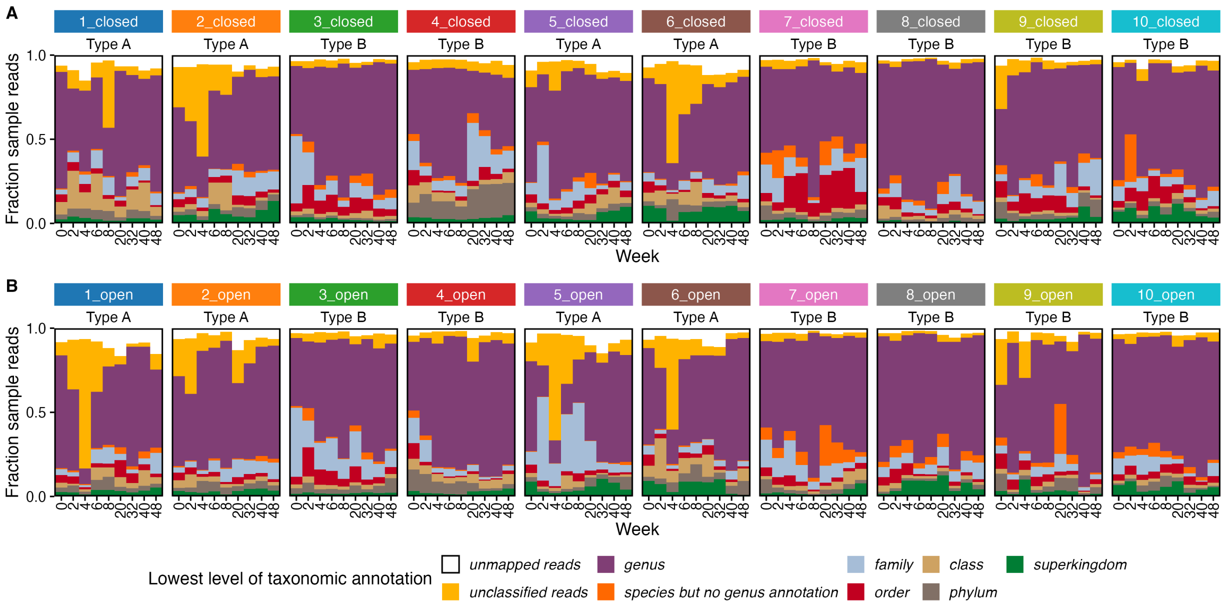

Fig. S2. Fraction of sample reads annotated at the genus level or, when not possible, to the lowest available taxonomic rank.

(**A**) Closed mesocosms 1-10. (**B**) Open mesocosms 1-10. Community type (A or B) for each mesocosm is indicated; see main text for details. Reads in orange represent cases where the top-scoring match in the NCBI non-redundant protein database included species-rank annotation but no genus-rank annotation.

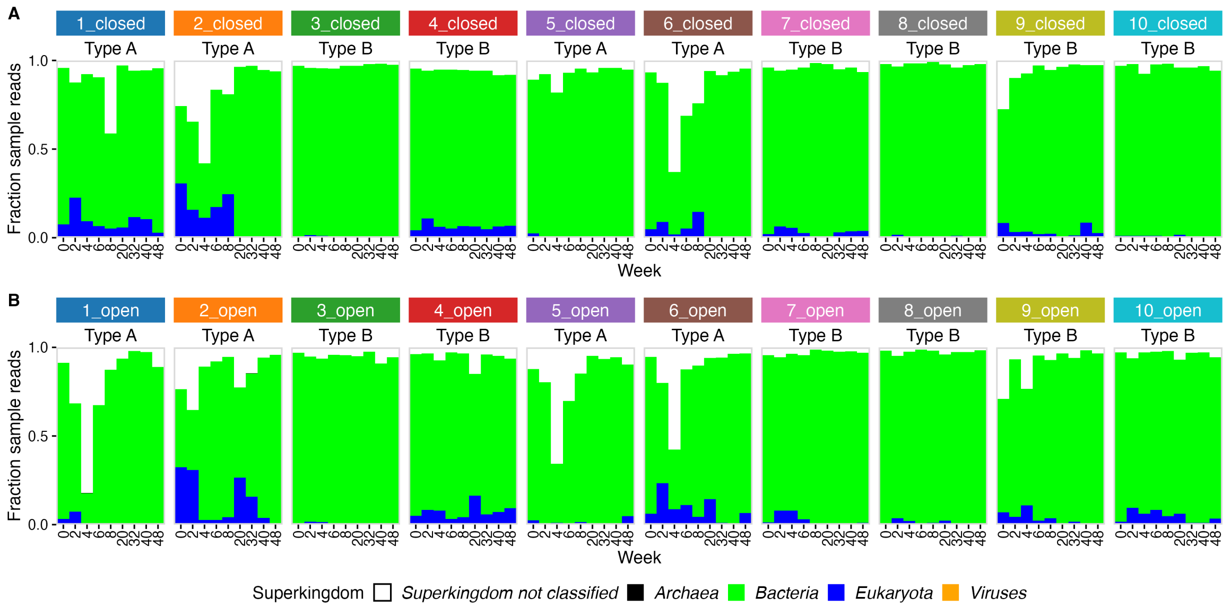

Fig. S3. Fraction of sample reads annotated at superkingdom rank using RAT. (**A**) Closed mesocosms 1-10. (**B**) Open mesocosms 1-10. See Methods for details.

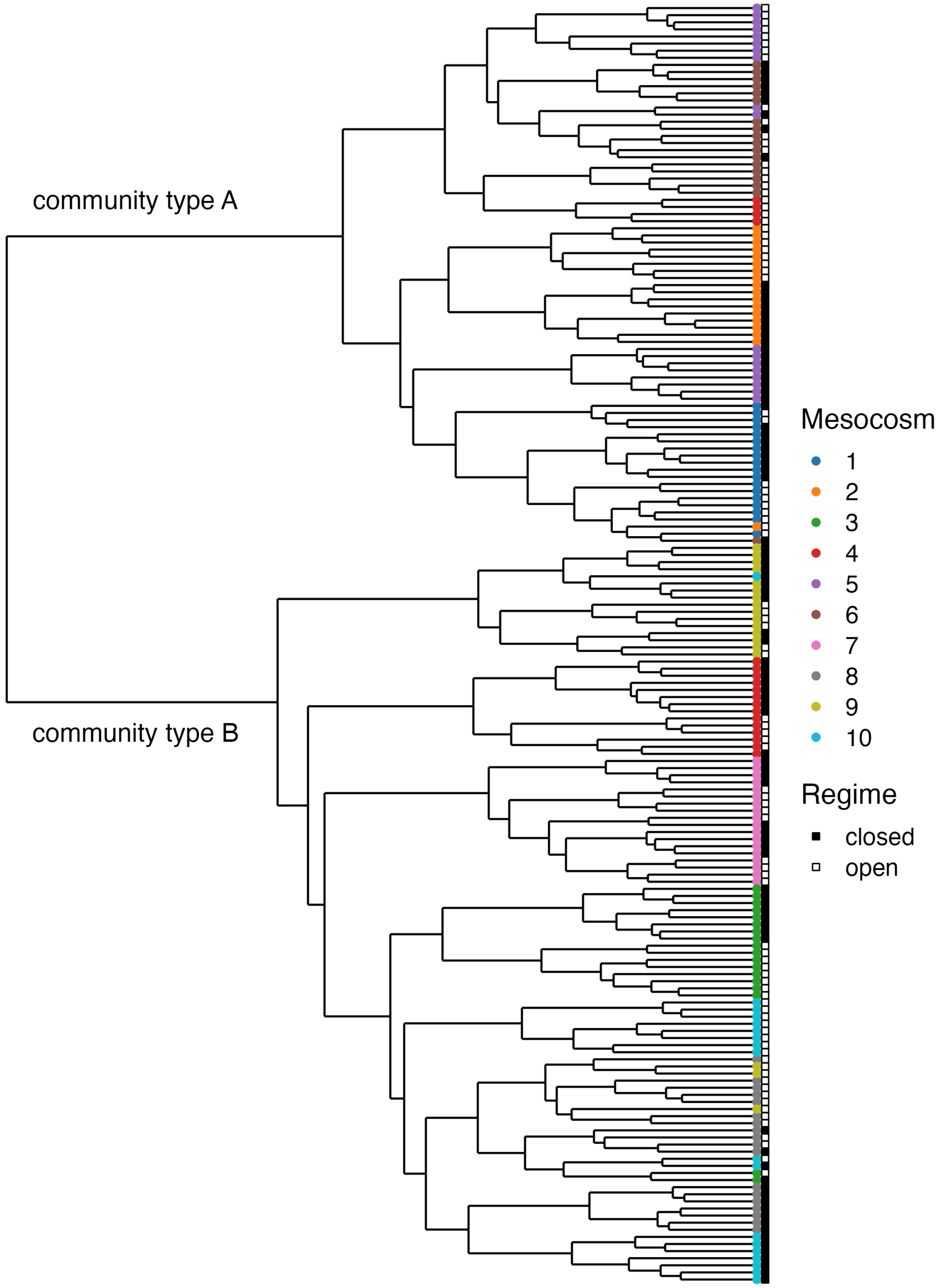

Fig. S4. Hierarchical clustering of mesocosm samples using Ward's method on Aitchison distances derived from genus-level abundances. Samples cluster into two distinct community types, consistent with the PCA shown in Fig. 1D of the main text (see Methods for details).

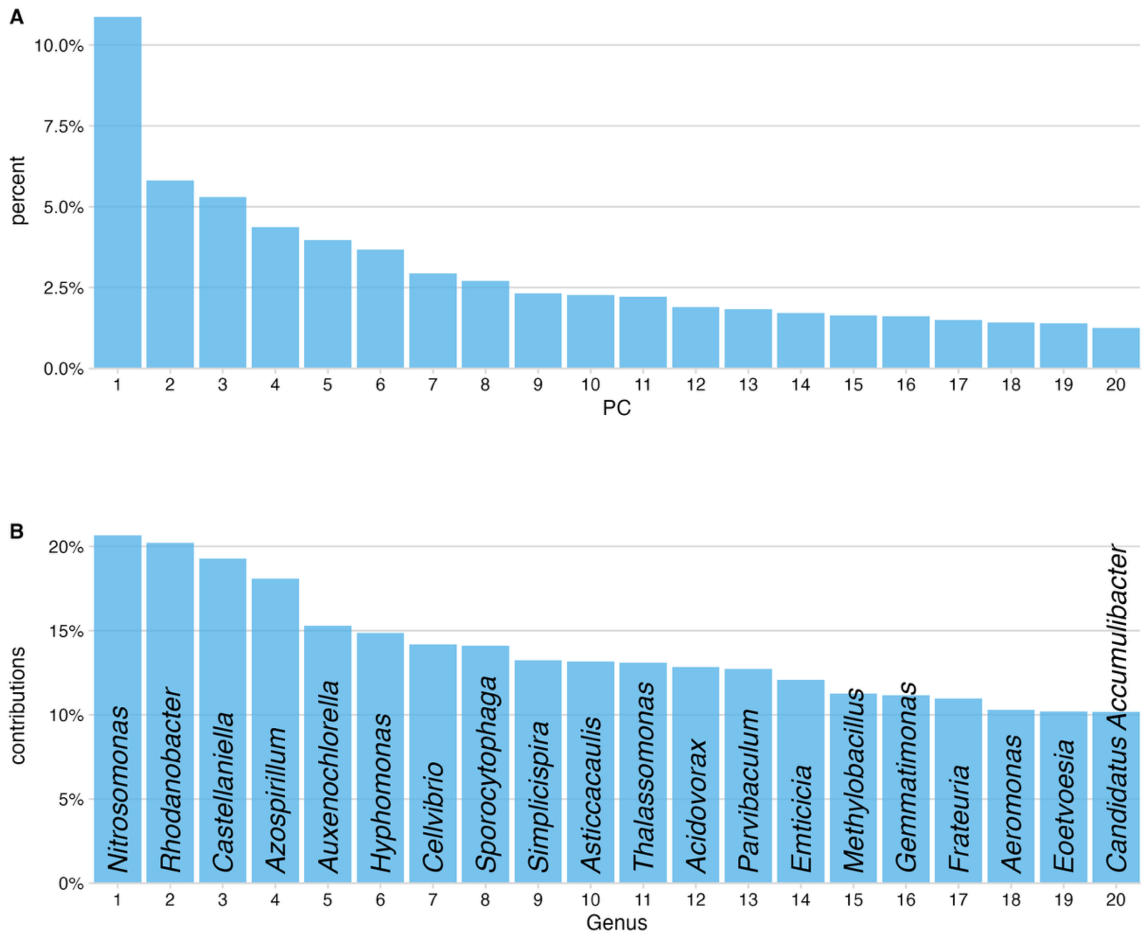

Fig. S5. PCA variance and top contributing genera.
(**A**) Variance explained by the top 20 principal components in the PCA shown in Fig. 1d (main text). (**B**) Top 20 genera contributing to PC1 and PC2.

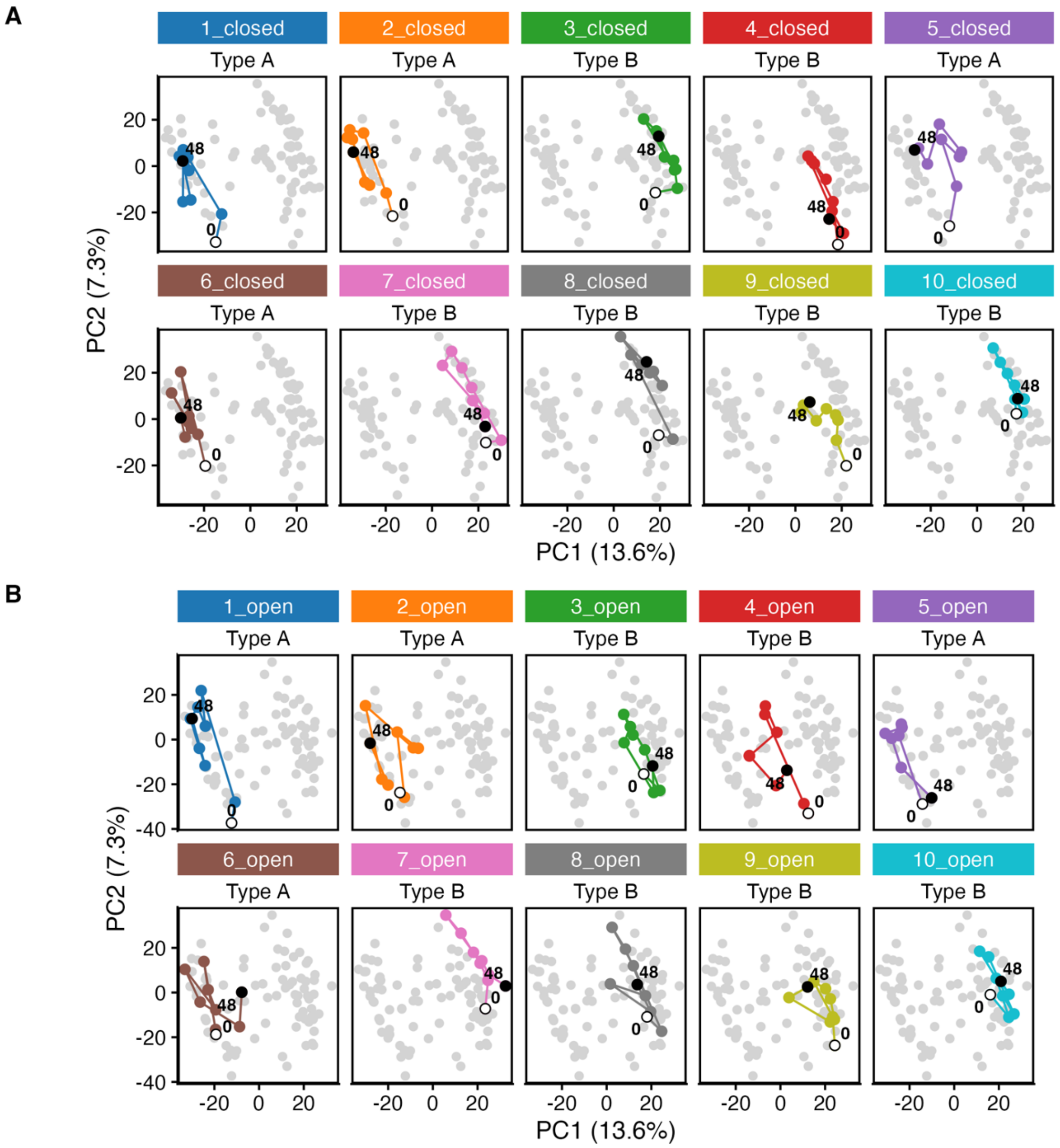

Fig. S6. Community trajectories in closed and open mesocosms over 48 weeks.
(**A**) PCA of closed mesocosms (1_closed–10_closed), taxonomically profiled at genus rank. Consecutive samples are connected by lines per mesocosm, with open and filled symbols indicating week 0 and week 48, respectively. (**B**) Same as (A) for open mesocosms.

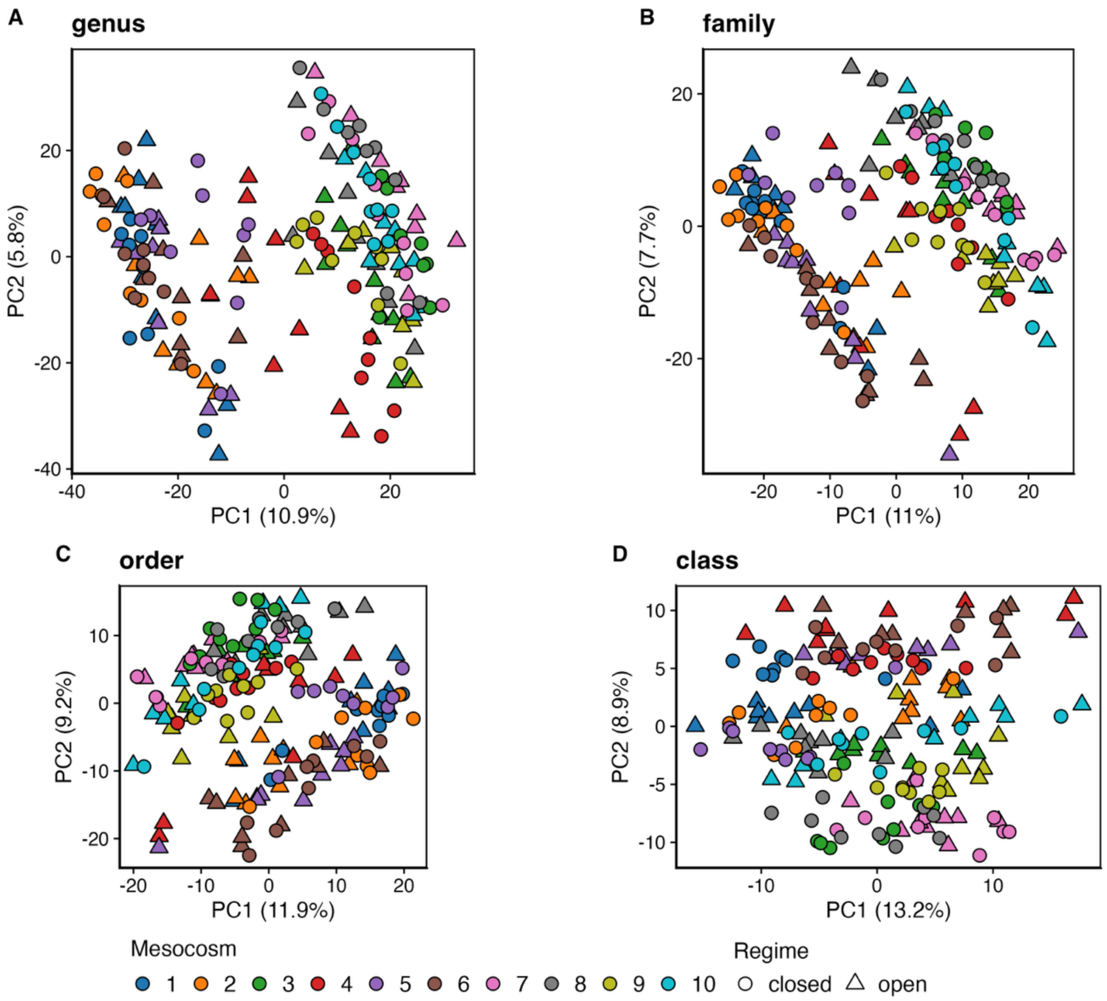

Fig. S7. Community profiling at higher taxonomic ranks preserves separation into two community types.

(**A**) Identical to Fig. 1d (main text), included for reference. (**B–D**) PCA based on reads annotated at family (B), order (C), and class (D) ranks, respectively.

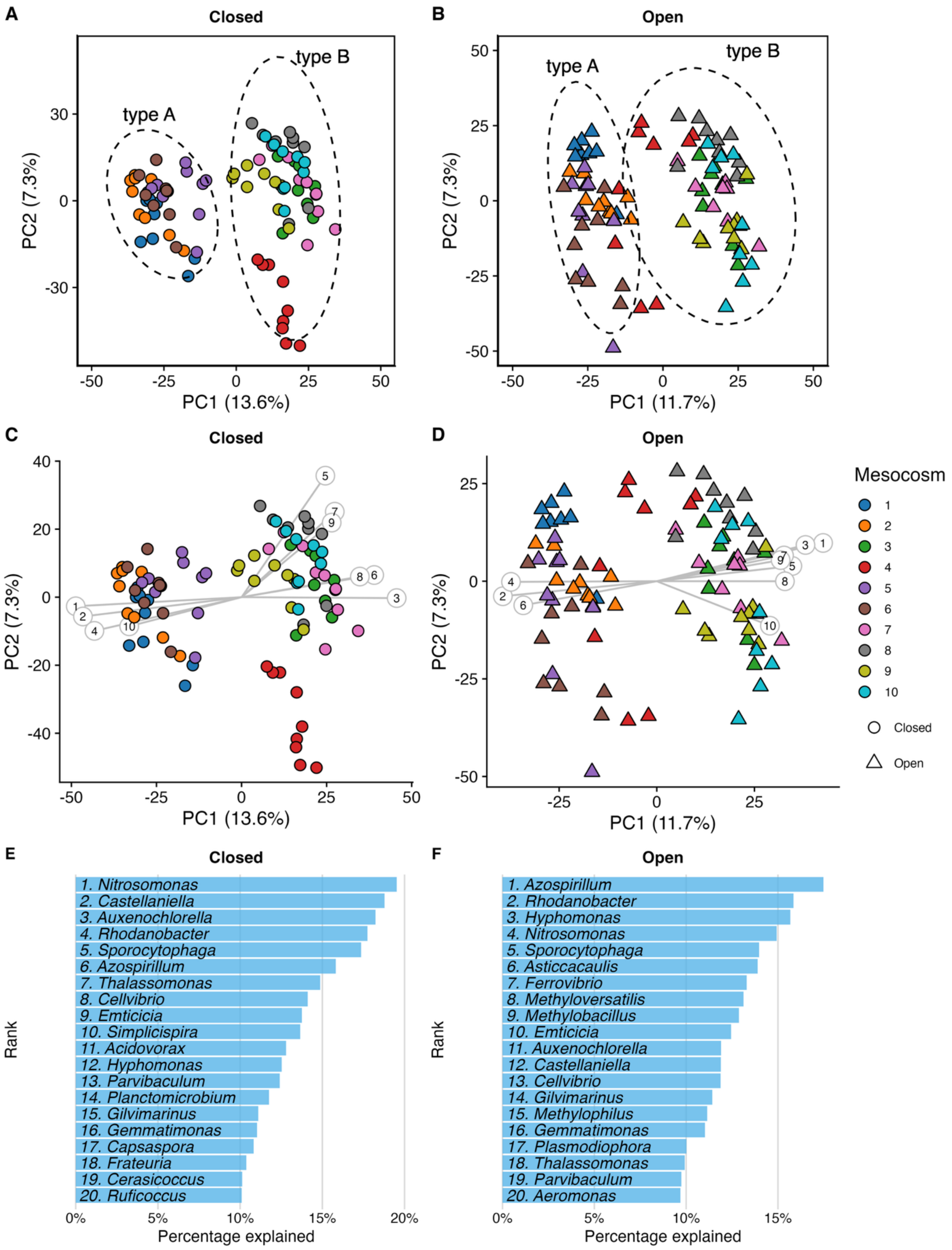
A
Closed
type A
type B
PC1 (13.6%)
PC2 (7.3%)
B
Open
type A
type B
PC1 (11.7%)
PC2 (7.3%)
C
Closed
PC1 (13.6%)
PC2 (7.3%)
D
Open
PC1 (11.7%)
PC2 (7.3%)
Mesocosm
Closed
Open
E
Closed
1. Nitrosomonas
2. Castellaniella
3. Auxenochlorella
4. Rhodanobacter
5. Sporocytophaga
6. Azospirillum
7. Thalassomonas
8. Cellvibrio
9. Emticicia
10. Simplicispira
11. Acidovorax
12. Hyphomonas
13. Parvibaculum
14. Planctomicrobium
15. Gilvimarinus
16. Gemmatimonas
17. Capsaspora
18. Frateuria
19. Cerasicoccus
20. Ruficoccus
Rank
Percentage explained
F
Open
1. Azospirillum
2. Rhodanobacter
3. Hyphomonas
4. Nitrosomonas
5. Sporocytophaga
6. Asticcacaulis
7. Ferrovibrio
8. Methyloversatilis
9. Methylobacillus
10. Emticicia
11. Auxenochlorella
12. Castellaniella
13. Cellvibrio
14. Gilvimarinus
15. Methylophilus
16. Gemmatimonas
17. Plasmodiophora
18. Thalassomonas
19. Parvibaculum
20. Aeromonas
Rank
Percentage explained

Fig. S8 (previous page). Separate PCA of closed and open mesocosms.
(**A, B**) Principal Component Analysis performed separately for closed (A) and open (B) mesocosms, in contrast to the combined PCA in the main text; community types remain separated along PC1 in both datasets. Ellipses indicate 95% confidence intervals for type A (mesocosms 1, 2, 5, 6) and type B (mesocosms 3, 4, 7–10). (**C, D**) Same analyses as (A, B), respectively, but highlighting the top 10 principal components shown in panels (E) and (F). (**E, F**) Identities and variance explained by the top 20 components in closed (E) and open (F) mesocosms.

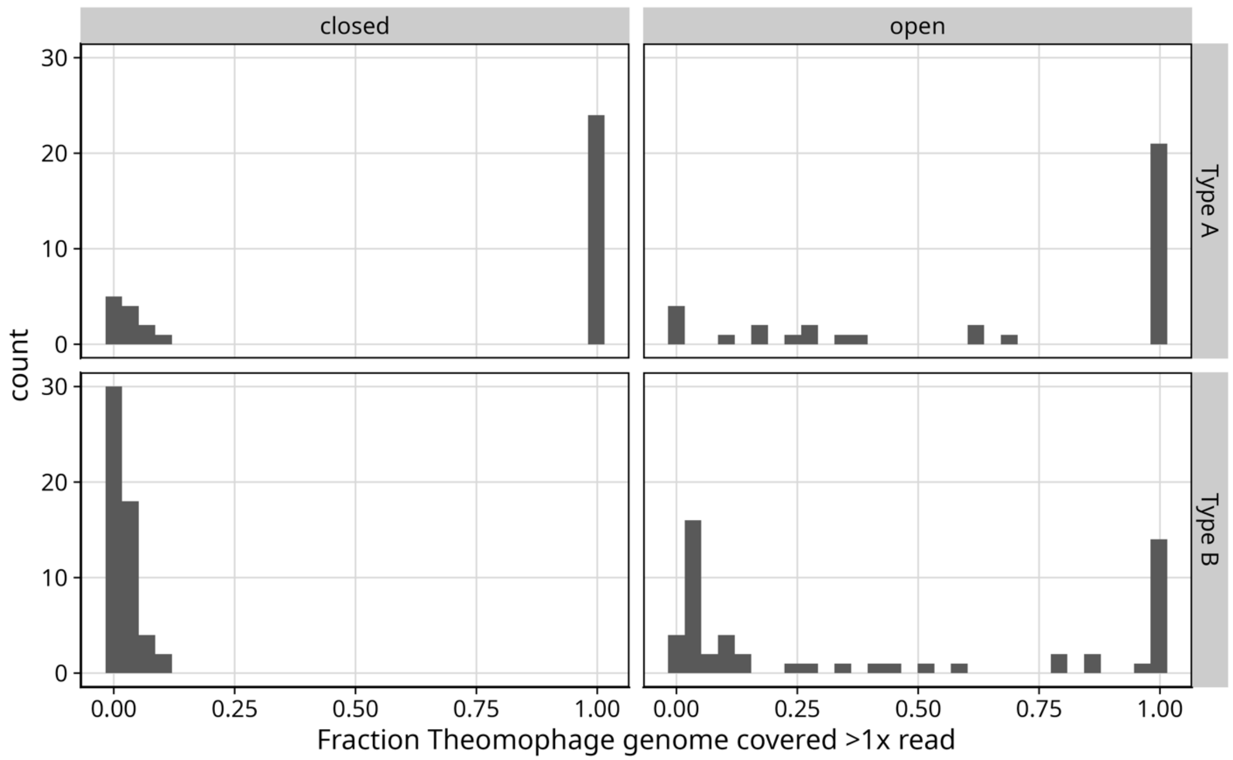

Fig. S9. Theomophage genome coverage across samples by community type and regime. Histograms show the number of samples at a given horizontal coverage (number of nucleotides with ≥1 read mapped) of the Theomophage genome in closed and open samples. Low horizontal coverage indicates absence of Theomophage in type-B communities in the closed experimental regime.

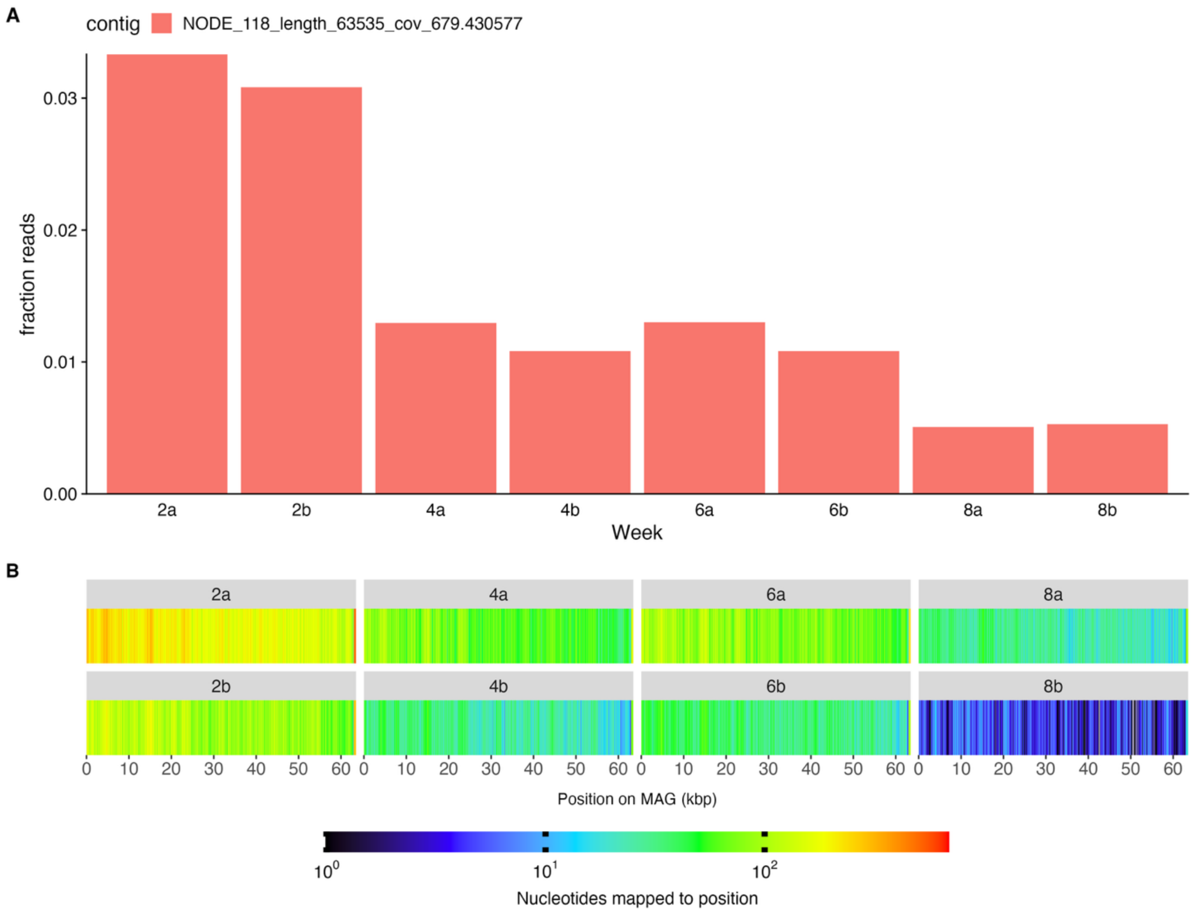

Fig. S10. Consistent detection and complete genome coverage of Theomophage in MGE cocktail samples.

(**A**) Relative abundance (fraction of total sample reads) of Theomophage contig NODE_118 in MGE cocktail samples from week 0, 2, 4 and 8. Each sample was sequenced twice, as indicated with letters a, b. (**B**) Read coverage profile for the Theomophage MAG showing detection of the complete genome in all cocktail samples.

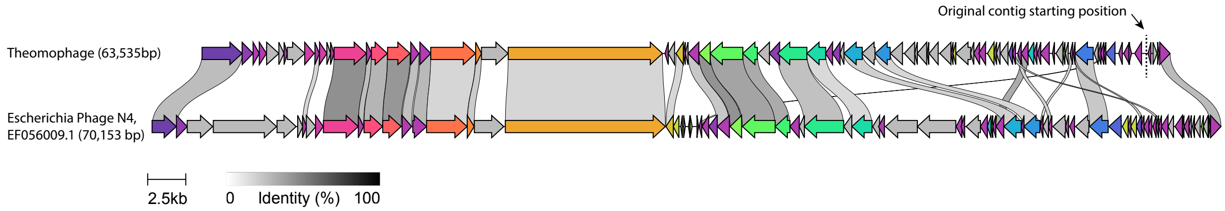

Fig. S11. Comparative genome architecture of Theomophage and *Escherichia* phage N4. Theomophage shows conserved synteny characteristic of the *Schitoviridae*, with a gene arrangement closely matching that of the prototypical *Schitovirus Escherichia* phage N4. Genomes were reoriented to begin at the terminase large subunit and are color-coded by orthogroup, with connecting links indicating a pairwise protein similarity exceeding a 24% identity threshold. The original Theomophage genome starting position is indicated with a dashed line.

Terminase Large Subunit

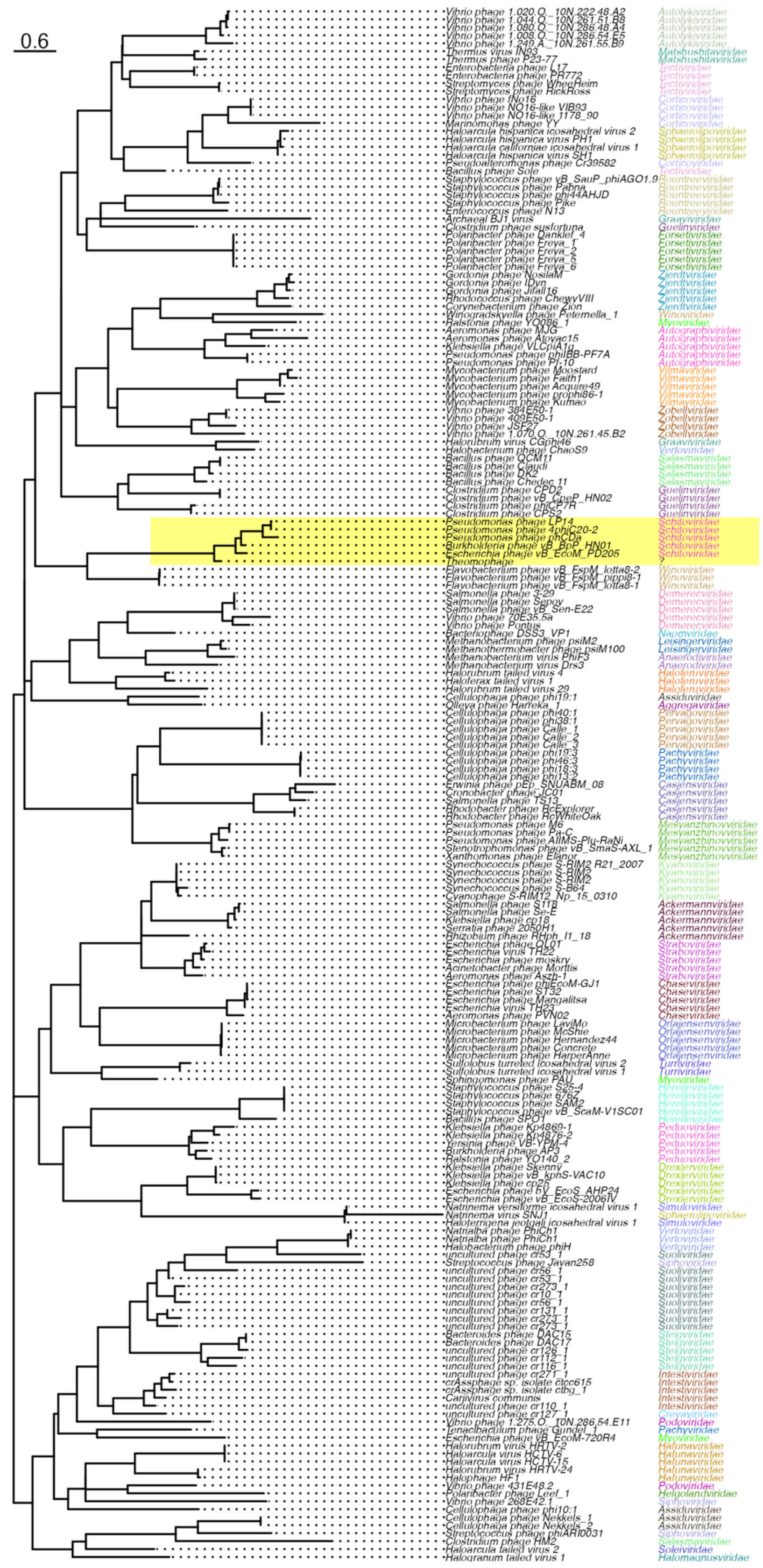

Fig. S12 (previous page) Unrooted phylogenetic tree of terminase large subunit proteins. The tree was constructed using five randomly selected representatives from each phage family in the INPHARED dataset, together with the terminase large subunit protein from Theomophage (see Methods for details). Theomophage clusters with members of the *Schitoviridae* family (highlighted in yellow). Node labels indicate species name and phage family (color-coded).

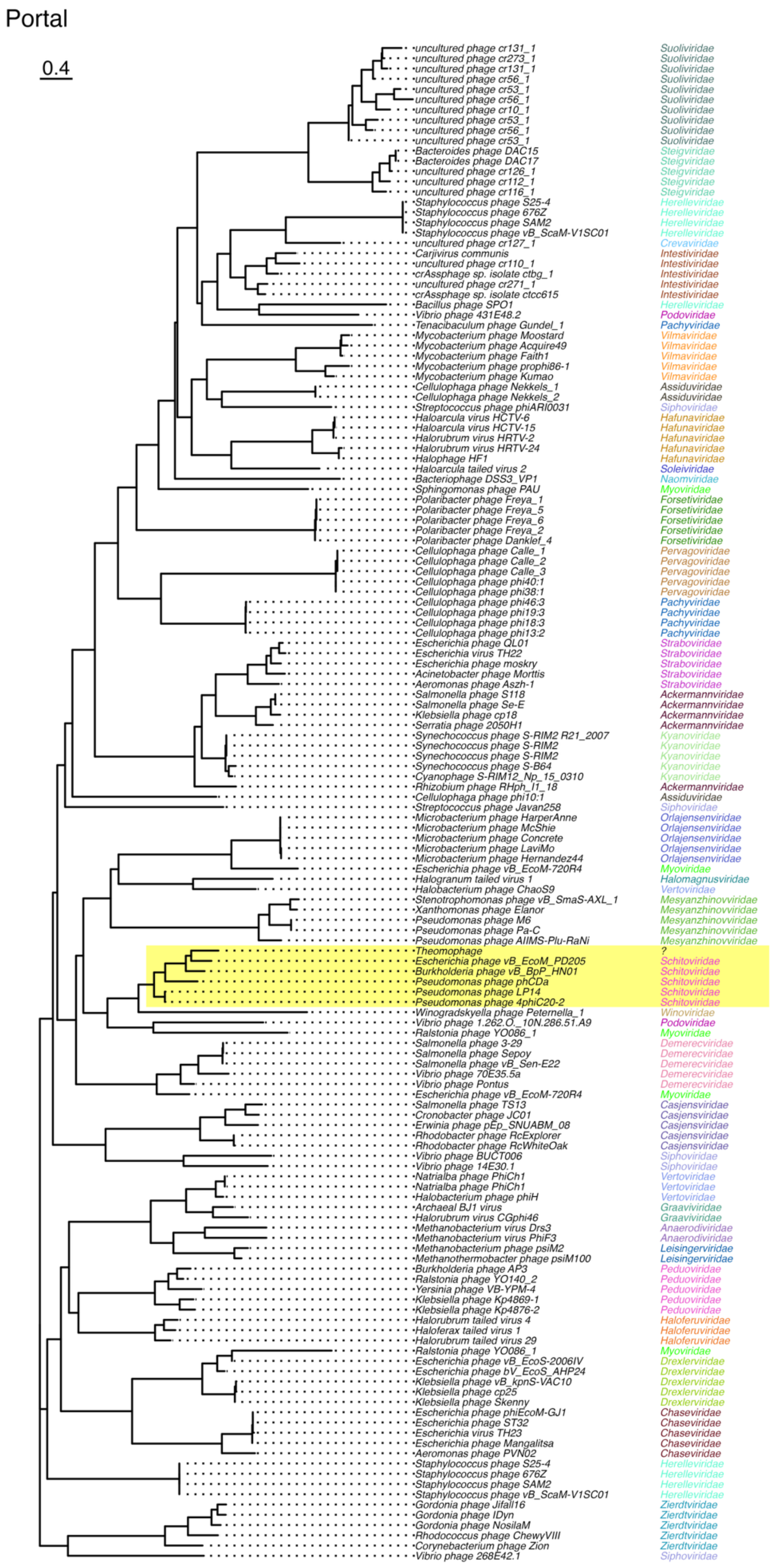
Portal
0.4
uncultured phage cr131_1 Suoliviridae
uncultured phage cr273_1 Suoliviridae
uncultured phage cr131_1 Suoliviridae
uncultured phage cr56_1 Suoliviridae
uncultured phage cr53_1 Suoliviridae
uncultured phage cr56_1 Suoliviridae
uncultured phage cr10_1 Suoliviridae
uncultured phage cr53_1 Suoliviridae
uncultured phage cr56_1 Suoliviridae
uncultured phage cr53_1 Suoliviridae
Bacteroides phage DAC15 Steigviridae
Bacteroides phage DAC17 Steigviridae
uncultured phage cr126_1 Steigviridae
uncultured phage cr112_1 Steigviridae
uncultured phage cr116_1 Steigviridae
Staphylococcus phage S25-4 Herelleviridae
Staphylococcus phage 676Z Herelleviridae
Staphylococcus phage SAM2 Herelleviridae
Staphylococcus phage vB_ScaM-V1SC01 Herelleviridae
uncultured phage cr127_1 Crevaviridae
Carjivirus communis Intestiviridae
uncultured phage cr110_1 Intestiviridae
crAssphage sp. isolate ctbg_1 Intestiviridae
uncultured phage cr271_1 Intestiviridae
crAssphage sp. isolate ctcc615 Intestiviridae
Bacillus phage SPO1 Herelleviridae
Vibrio phage 431E48.2 Podoviridae
Tenacibaculum phage Gundel_1 Pachyviridae
Mycobacterium phage Moostard Vilmaviridae
Mycobacterium phage Acquire49 Vilmaviridae
Mycobacterium phage Faith1 Vilmaviridae
Mycobacterium phage prophi86-1 Vilmaviridae
Mycobacterium phage Kumao Vilmaviridae
Cellulophaga phage Nekkels_1 Assiduviridae
Cellulophaga phage Nekkels_2 Assiduviridae
Streptococcus phage phiARI0031 Siphoviridae
Haloarcula virus HCTV-6 Hafunaviridae
Haloarcula virus HCTV-15 Hafunaviridae
Halorubrum virus HRTV-2 Hafunaviridae
Halorubrum virus HRTV-24 Hafunaviridae
Halophage HF1 Hafunaviridae
Haloarcula tailed virus 2 Soleiviridae
Bacteriophage DSS3_VP1 Naomviridae
Sphingomonas phage PAU Myoviridae
Polaribacter phage Freya_1 Forsetiviridae
Polaribacter phage Freya_5 Forsetiviridae
Polaribacter phage Freya_6 Forsetiviridae
Polaribacter phage Freya_2 Forsetiviridae
Polaribacter phage Danklef_4 Forsetiviridae
Cellulophaga phage Calle_1 Pervagoviridae
Cellulophaga phage Calle_2 Pervagoviridae
Cellulophaga phage Calle_3 Pervagoviridae
Cellulophaga phage phi40:1 Pervagoviridae
Cellulophaga phage phi38:1 Pervagoviridae
Cellulophaga phage phi46:3 Pachyviridae
Cellulophaga phage phi19:3 Pachyviridae
Cellulophaga phage phi18:3 Pachyviridae
Cellulophaga phage phi13:2 Pachyviridae
Escherichia phage QL01 Straboviridae
Escherichia virus TH22 Straboviridae
Escherichia phage moskry Straboviridae
Acinetobacter phage Morttis Straboviridae
Aeromonas phage Aszh-1 Straboviridae
Salmonella phage S118 Ackermannviridae
Salmonella phage Se-E Ackermannviridae
Klebsiella phage cp18 Ackermannviridae
Serratia phage 2050H1 Ackermannviridae
Synechococcus phage S-RIM2 R21_2007 Kyanoviridae
Synechococcus phage S-RIM2 Kyanoviridae
Synechococcus phage S-RIM2 Kyanoviridae
Synechococcus phage S-B64 Kyanoviridae
Cyanophage S-RIM12_Np_15_0310 Kyanoviridae
Rhizobium phage RHph_I1_18 Ackermannviridae
Cellulophaga phage phi10:1 Assiduviridae
Streptococcus phage Javan258 Siphoviridae
Microbacterium phage HarperAnne Orlajensenviridae
Microbacterium phage McShie Orlajensenviridae
Microbacterium phage Concrete Orlajensenviridae
Microbacterium phage LaviMo Orlajensenviridae
Microbacterium phage Hernandez44 Orlajensenviridae
Escherichia phage vB_EcoM-720R4 Myoviridae
Halogranum tailed virus 1 Halomagnusviridae
Halobacterium phage ChaoS9 Vertoviridae
Stenotrophomonas phage vB_SmaS-AXL_1 Mesyanzhinovviridae
Xanthomonas phage Elanor Mesyanzhinovviridae
Pseudomonas phage M6 Mesyanzhinovviridae
Pseudomonas phage Pa-C Mesyanzhinovviridae
Pseudomonas phage AIIMS-Plu-RaNi Mesyanzhinovviridae
Theomophage ?
Escherichia phage vB_EcoM_PD205 Schitoviridae
Burkholderia phage vB_BpP_HN01 Schitoviridae
Pseudomonas phage phCDa Schitoviridae
Pseudomonas phage LP14 Schitoviridae
Pseudomonas phage 4phiC20-2 Schitoviridae
Winogradskyella phage Peternella_1 Winoviridae
Vibrio phage 1.262.O._10N.286.51.A9 Podoviridae
Ralstonia phage YO086_1 Myoviridae
Salmonella phage 3-29 Demerecviridae
Salmonella phage Sepoy Demerecviridae
Salmonella phage vB_Sen-E22 Demerecviridae
Vibrio phage 70E35.5a Demerecviridae
Vibrio phage Pontus Demerecviridae
Escherichia phage vB_EcoM-720R4 Myoviridae
Salmonella phage TS13 Casjensviridae
Cronobacter phage JC01 Casjensviridae
Erwinia phage pEp_SNUABM_08 Casjensviridae
Rhodobacter phage RcExplorer Casjensviridae
Rhodobacter phage RcWhiteOak Casjensviridae
Vibrio phage BUCT006 Siphoviridae
Vibrio phage 14E30.1 Siphoviridae
Natrialba phage PhiCh1 Vertoviridae
Natrialba phage PhiCh1 Vertoviridae
Halobacterium phage phiH Vertoviridae
Archaeal BJ1 virus Graaviviridae
Halorubrum virus CGphi46 Graaviviridae
Methanobacterium virus Drs3 Anaerodiviridae
Methanobacterium virus PhiF3 Anaerodiviridae
Methanobacterium phage psiM2 Leisingerviridae
Methanothermobacter phage psiM100 Leisingerviridae
Burkholderia phage AP3 Peduoviridae
Ralstonia phage YO140_2 Peduoviridae
Yersinia phage VB-YPM-4 Peduoviridae
Klebsiella phage Kp4869-1 Peduoviridae
Klebsiella phage Kp4876-2 Peduoviridae
Halorubrum tailed virus 4 Haloferuviridae
Haloferax tailed virus 1 Haloferuviridae
Halorubrum tailed virus 29 Haloferuviridae
Ralstonia phage YO086_1 Myoviridae
Escherichia phage vB_EcoS-2006IV Drexlerviridae
Escherichia phage bV_EcoS_AHP24 Drexlerviridae
Klebsiella phage vB_kpnS-VAC10 Drexlerviridae
Klebsiella phage cp25 Drexlerviridae
Klebsiella phage Skenny Drexlerviridae
Escherichia phage phiEcoM-GJ1 Chaseviridae
Escherichia phage ST32 Chaseviridae
Escherichia virus TH23 Chaseviridae
Escherichia phage Mangalitsa Chaseviridae
Aeromonas phage PVN02 Chaseviridae
Staphylococcus phage S25-4 Herelleviridae
Staphylococcus phage 676Z Herelleviridae
Staphylococcus phage SAM2 Herelleviridae
Staphylococcus phage vB_ScaM-V1SC01 Herelleviridae
Gordonia phage Jifall16 Zierdtviridae
Gordonia phage IDyn Zierdtviridae
Gordonia phage NosilaM Zierdtviridae
Rhodococcus phage ChewyVIII Zierdtviridae
Corynebacterium phage Zion Zierdtviridae
Vibrio phage 268E42.1 Siphoviridae

Fig. S13 (previous page) Unrooted phylogenetic tree of portal proteins.
The tree was constructed using five randomly selected representatives from each phage family in the INPHARED dataset, together with the portal protein from Theomophage (see Methods for details). Theomophage clusters with members of the *Schitoviridae* family (highlighted in yellow). Node labels indicate species name and phage family (color-coded).

Major Capsid Protein

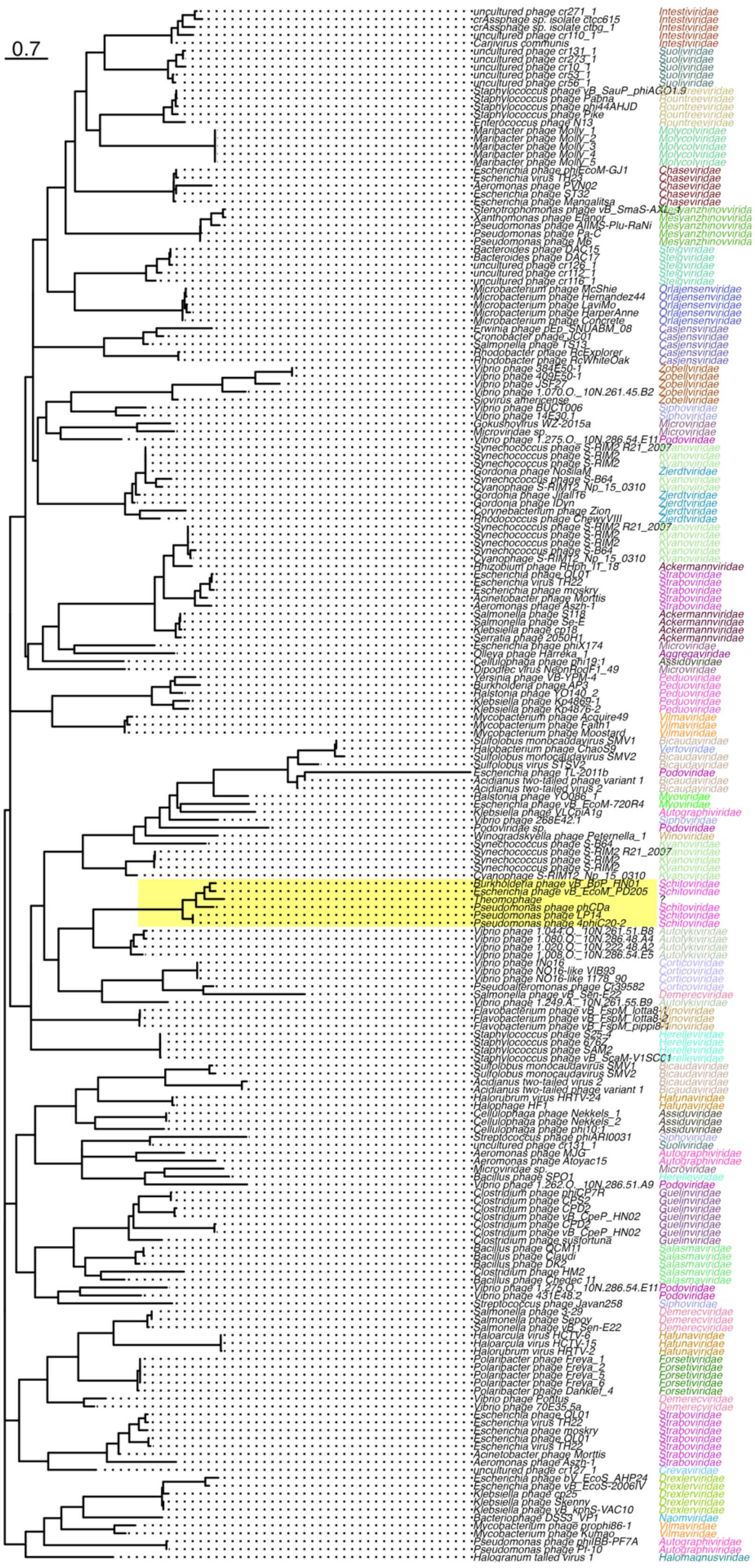

Fig. S14 (previous page) Unrooted phylogenetic tree of Major capsid proteins.
The tree was constructed using five randomly selected representatives from each phage family in the INPHARED dataset, together with the Theomophage major capsid protein from (see Methods for details). Theomophage clusters with members of the *Schitoviridae* family (highlighted in yellow). Node labels indicate species name and phage family (color-coded).

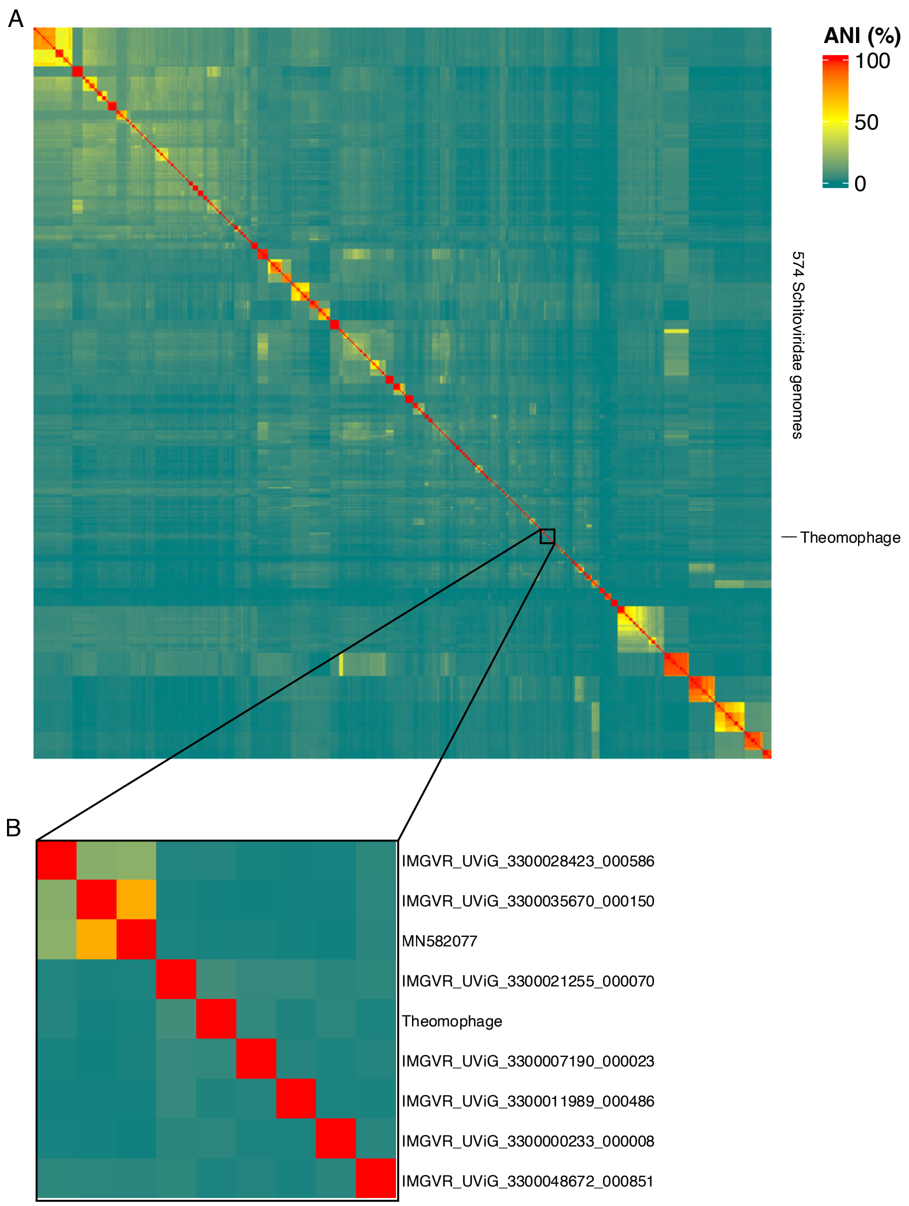

Fig. S15. **Genomic similarity of *Schitoviridae* genomes and Theomophage.**
(**A**) Heatmap of VIRIDIC-based pairwise similarity of 573 high-quality (>90% complete) *Schitoviridae* genomes and the Theomophage MAG NODE244. Theomophage shows only 8.98% similarity to known *Schitoviridae*, below the 20% subfamily demarcation threshold, supporting its designation as a novel subfamily. (**B**) inset showing location of Theomophage.

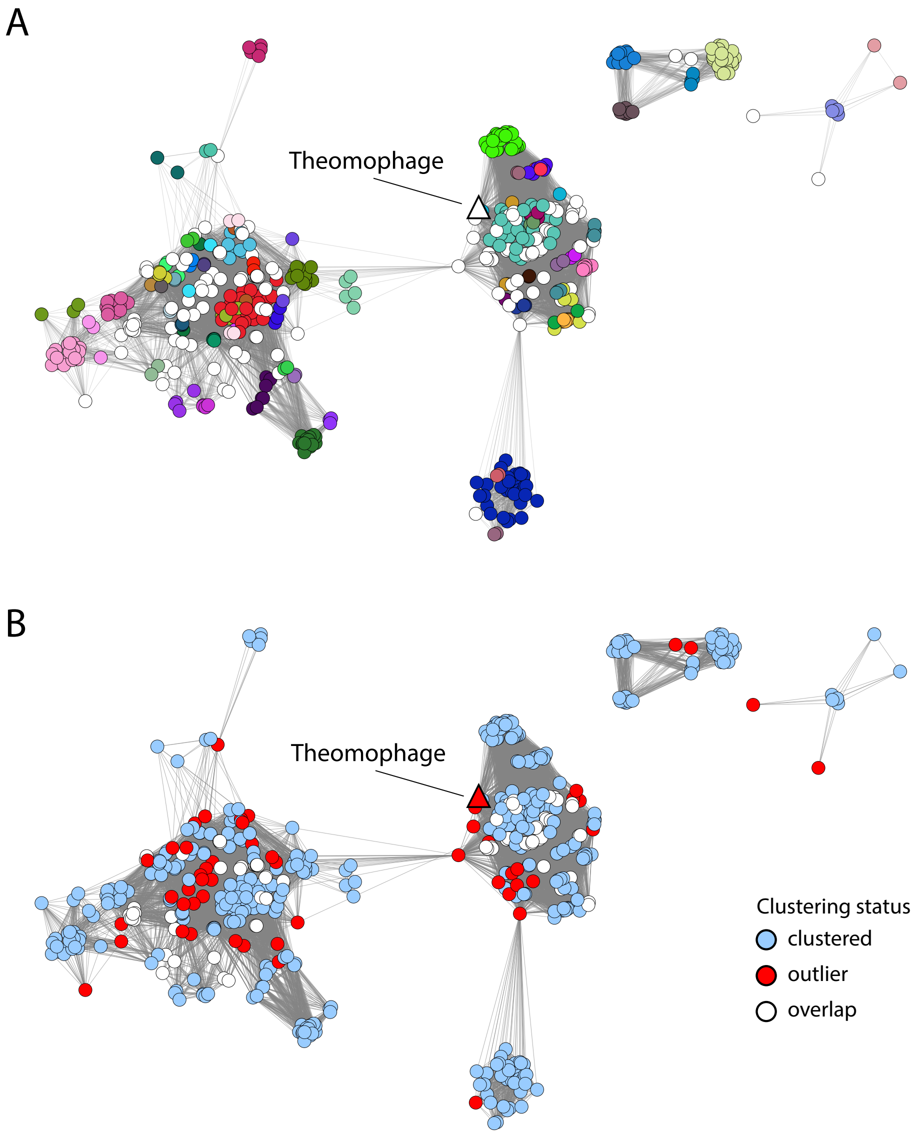

Fig. S16. Gene sharing network of 573 high-quality *Schitoviridae* genomes and Theomophage MAG NODE244.
(**A**) Network constructed with vConTACT2 using default parameters. Each node represents a viral genome and is colored by the assigned vConTACT2 viral cluster (VC), which reflects ICTV genus level taxonomy. Theomophage (triangle) does not cluster with any known *Schitoviridae* sequences, indicating it is not related at genus level. (**B**) Same network as in (A), but nodes colored by clustering status: genomes assigned to a single VC (blue), overlap belonging to multiple VCs (white), and outliers that do not group into any VC (red).

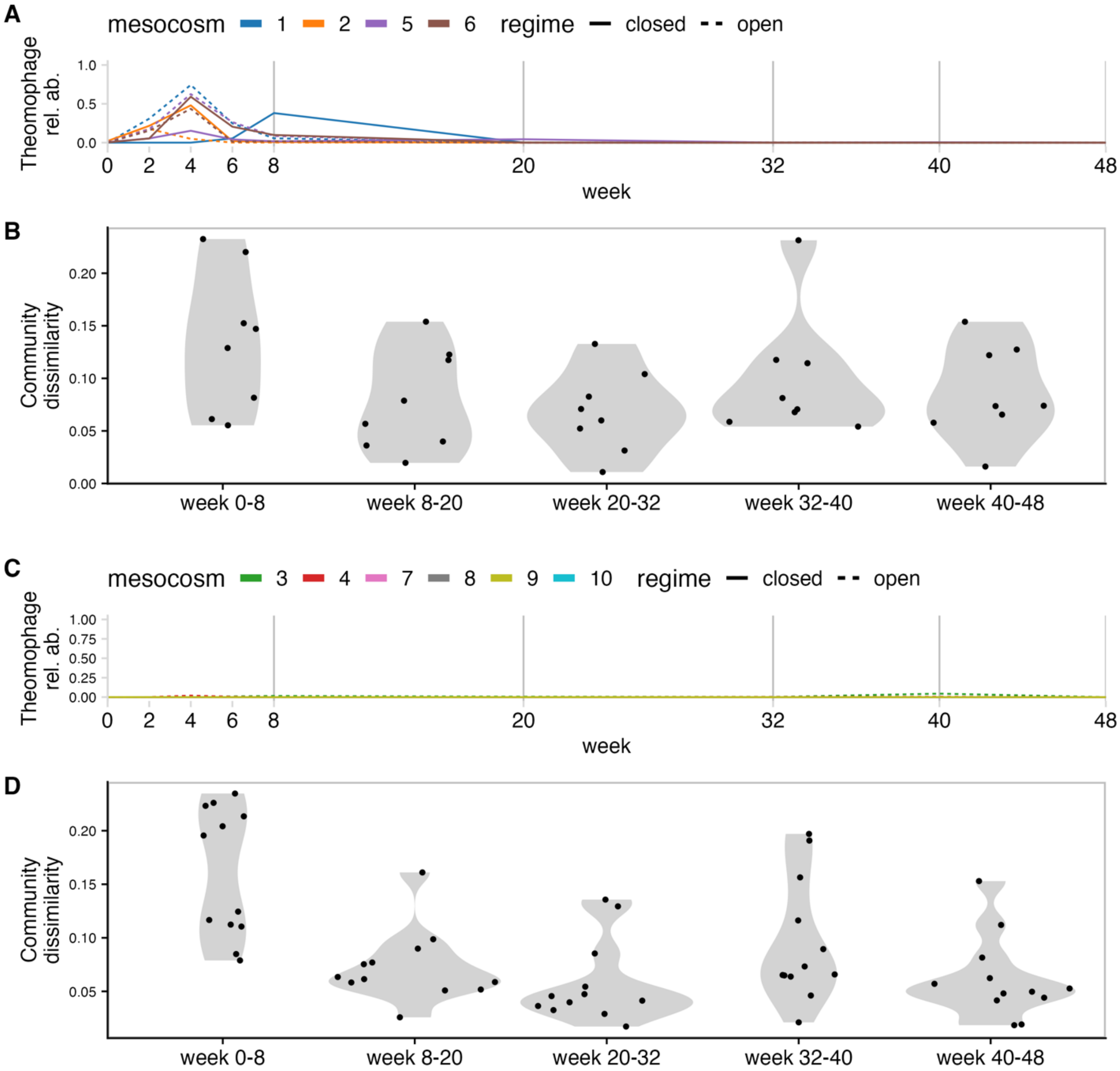

Figure S17. Theomophage outbreaks do not alter community composition.
(**A**) Theomophage abundance in mesocosms with outbreaks (closed and open 1,2,5,6; outbreaks observed between weeks 0-8). (**B**) Bray-Curtis dissimilarity of genus abundances for time points spanning Theomophage outbreaks (weeks 0–8) and without outbreaks (all other intervals). While dissimilarities appear higher during outbreaks, complex natural communities typically lose taxa when adapted to lab conditions[26], a trend also observed in mesocosms without outbreaks (D). (**C, D**) Same as (A, B), but for mesocosms without Theomophage outbreaks. Bray-Curtis dissimilarities were normalized for time interval length.

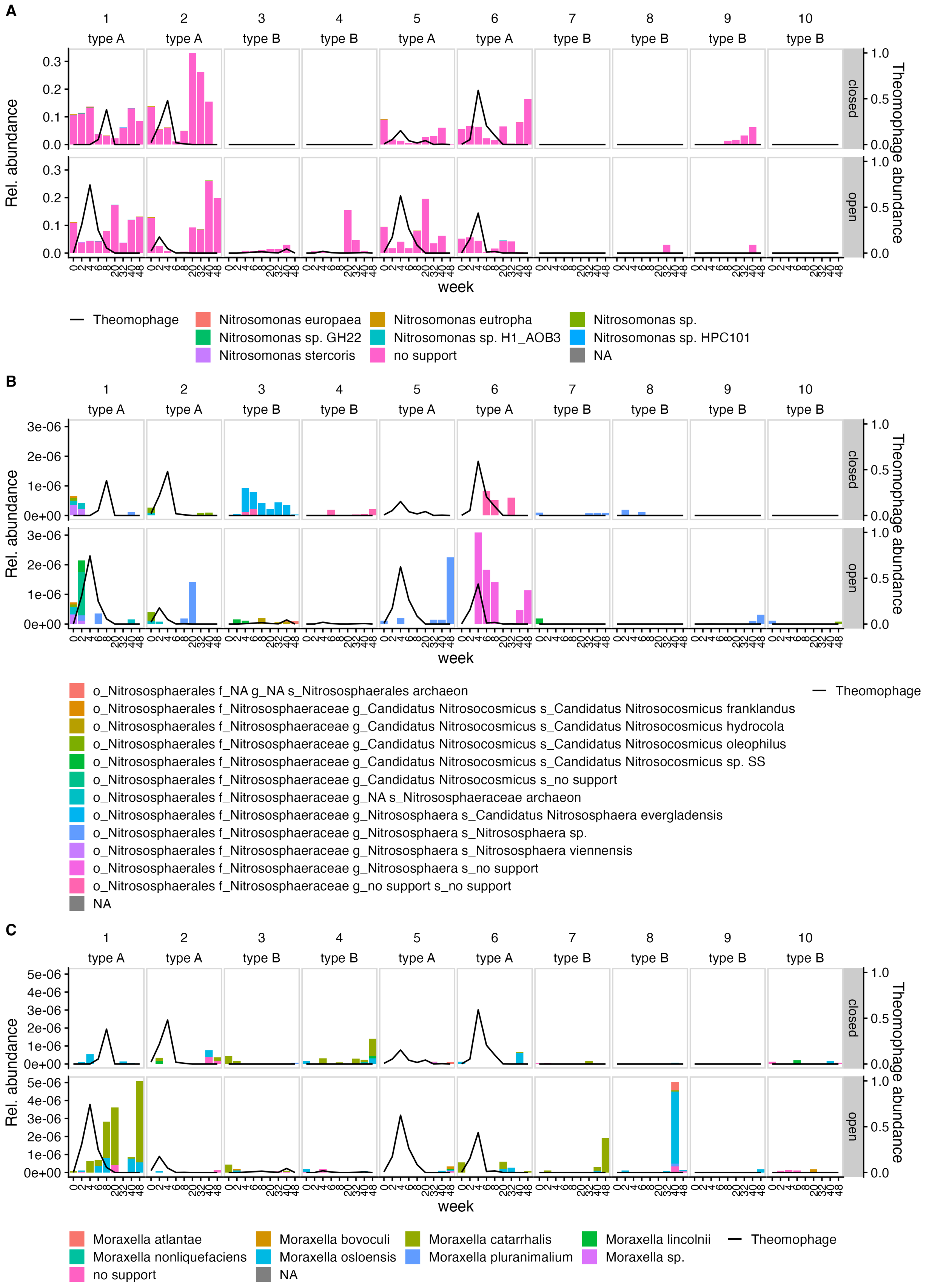

A
1
2
3
4
5
6
7
8
9
10
type A
type B
closed
open
Rel. abundance
Theomophage abundance
week
Theomophage
Nitrosomonas europaea
Nitrosomonas eutropha
Nitrosomonas sp.
Nitrosomonas sp. GH22
Nitrosomonas sp. H1_AOB3
Nitrosomonas sp. HPC101
Nitrosomonas stercoris
no support
NA
B
o_Nitrososphaerales f_NA g_NA s_Nitrososphaerales archaeon
o_Nitrososphaerales f_Nitrososphaeraceae g_Candidatus Nitrosocosmicus s_Candidatus Nitrosocosmicus franklandus
o_Nitrososphaerales f_Nitrososphaeraceae g_Candidatus Nitrosocosmicus s_Candidatus Nitrosocosmicus hydrocola
o_Nitrososphaerales f_Nitrososphaeraceae g_Candidatus Nitrosocosmicus s_Candidatus Nitrosocosmicus oleophilus
o_Nitrososphaerales f_Nitrososphaeraceae g_Candidatus Nitrosocosmicus s_Candidatus Nitrosocosmicus sp. SS
o_Nitrososphaerales f_Nitrososphaeraceae g_Candidatus Nitrosocosmicus s_no support
o_Nitrososphaerales f_Nitrososphaeraceae g_NA s_Nitrososphaeraceae archaeon
o_Nitrososphaerales f_Nitrososphaeraceae g_Nitrososphaera s_Candidatus Nitrososphaera evergladensis
o_Nitrososphaerales f_Nitrososphaeraceae g_Nitrososphaera s_Nitrososphaera sp.
o_Nitrososphaerales f_Nitrososphaeraceae g_Nitrososphaera s_Nitrososphaera viennensis
o_Nitrososphaerales f_Nitrososphaeraceae g_Nitrososphaera s_no support
o_Nitrososphaerales f_Nitrososphaeraceae g_no support s_no support
NA
Theomophage
C
Moraxella atlantae
Moraxella bovoculi
Moraxella catarrhalis
Moraxella lincolnii
Theomophage
Moraxella nonliquefaciens
Moraxella osloensis
Moraxella pluranimalium
Moraxella sp.
no support
NA

Fig. S18 (previous page). Abundance of putative Theomophage hosts across 10 closed and open compost mesocosms.

Host predictions were made using CRISPR spacer matching against the SpacerDB global spacer database and genome-based host prediction via iPHoP (see Methods and **Table S8-S10**). Theomophage abundance is shown as a black line (secondary y-axis). (**A**) *Nitrosomonas* species, identified as a candidate host based on six spacer matches with 2-3 mismatches in SpacerDB, providing weak evidence of a host relationship. However, *Nitrosomonas* was consistently associated with type-A communities where Theomophage outbreaks occurred (see Fig.1B-E) and was detected in 2 of the 5 mesocosms where Theomophage invaded (3_open and 4_open). Note that no CRISPR spacers targeting Theomophage were found in *Nitrosomonas* sequences from the compost metagenomes. (**B**) Taxa within the *Nitrososphaerales* order, predicted as a candidate host by iPHoP. (**C**) *Moraxella* species. A single spacer with one mismatch was found in SpacerDB, originating from a wastewater-associated *Moraxella* sequence. Both *Moraxella* and *Nitrososphaerales* were detected at very low abundance (0.000509% and 0.000309% of sample reads, respectively), making them unlikely hosts for the abundant Theomophage. However, since metagenomes were collected at the end of a two-week growth cycle, it remains possible that active phage predation led to host population collapse, reducing their population sizes to near the detection threshold at the time of sampling.

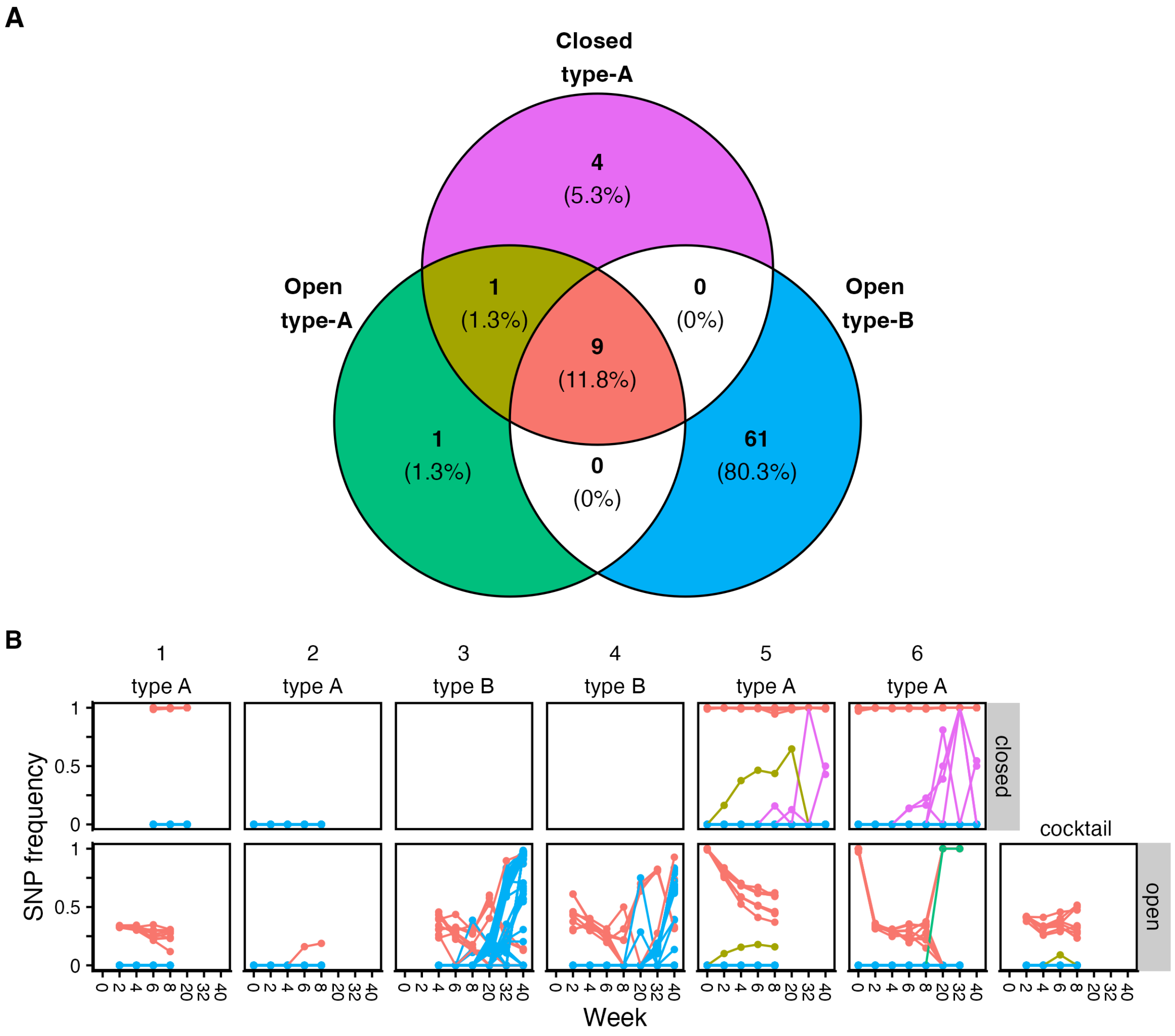

Fig. S19. Theomophage SNP variation and allele dynamics across communities.
(**A**) Venn diagram of 76 Theomophage SNPs detected across native (type-A) and novel (type-B) communities in closed and open regimes. Theomophage was not detected in type-B communities in the closed regime. (**B**) Allele frequency trajectories, with colors corresponding to (A). Olive and pink SNPs represent microdiversity within genotype G2 in mesocosms 5_closed, 6_closed and 5_open. The olive SNP is unique to community 5 and observed in both regimes. The green SNP is unique to 6_open.

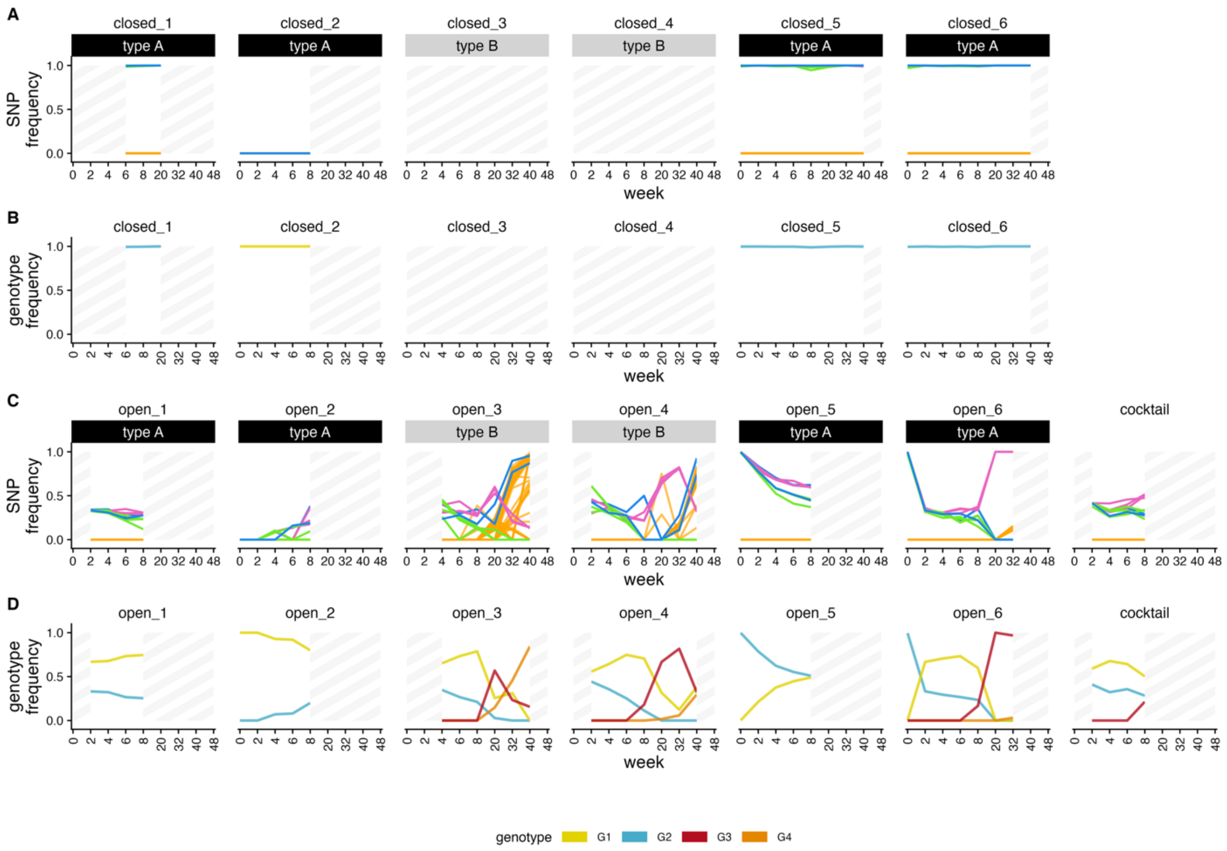

Fig. S20. More sensitive SNP analysis reveals additional migration events between open mesocosms.

Using the same approach as in Figure 4 of the main text, but with less strict variant filtering reveals that all nine SNPs defining genotype G2 were present at week 8 in mesocosm 2_open. Genotype G4 was also detected in 6_open at week 32, indicating migration to this mesocosm. SNP and genotype colors match Fig.4 and Table 1 of the main text. (**A, C**) SNP trajectories for closed and open mesocosms, respectively. (**B, D**) inferred genotype abundances for closed and open mesocosms, respectively.

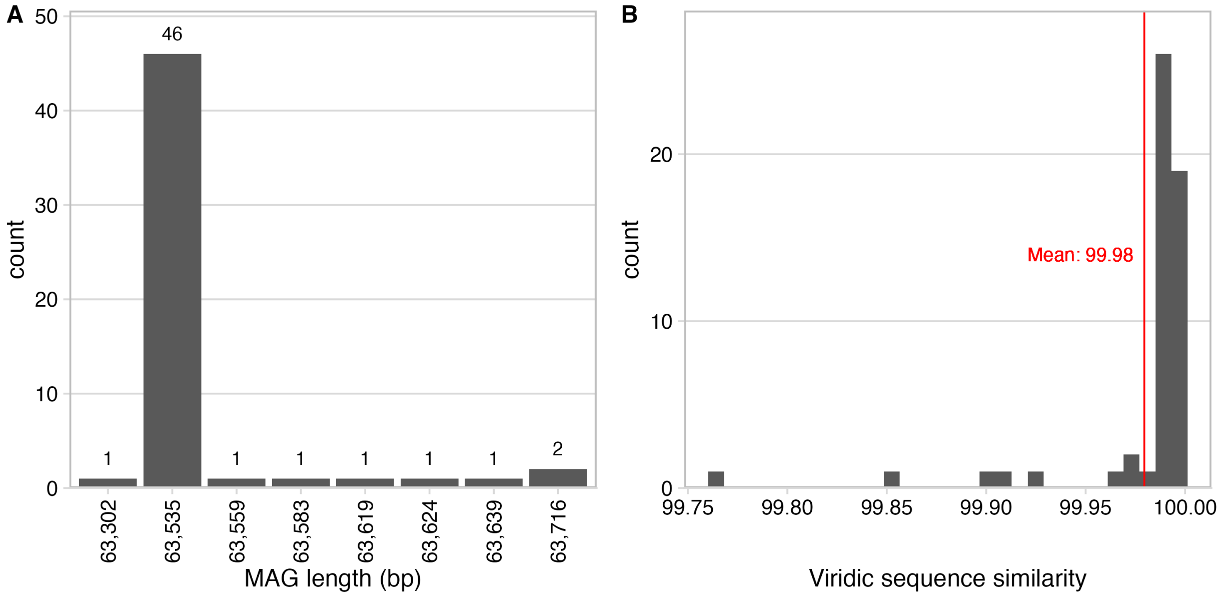

Fig. S21. Length and sequence similarity of Theomophage contigs assembled from different metagenomes.

(**A**) Length distribution of Theomophage contigs >60kb from 170 separately assembled metagenomes. Length of assembled contigs was highly consistent. Alignment and read pileup inspection (not shown) indicated contigs >63,535 bp are chimeric assemblies of multiple Theomophage genotypes. (**B**) Sequence similarity of contigs from (A), calculated with VIRIDIC (see Methods).

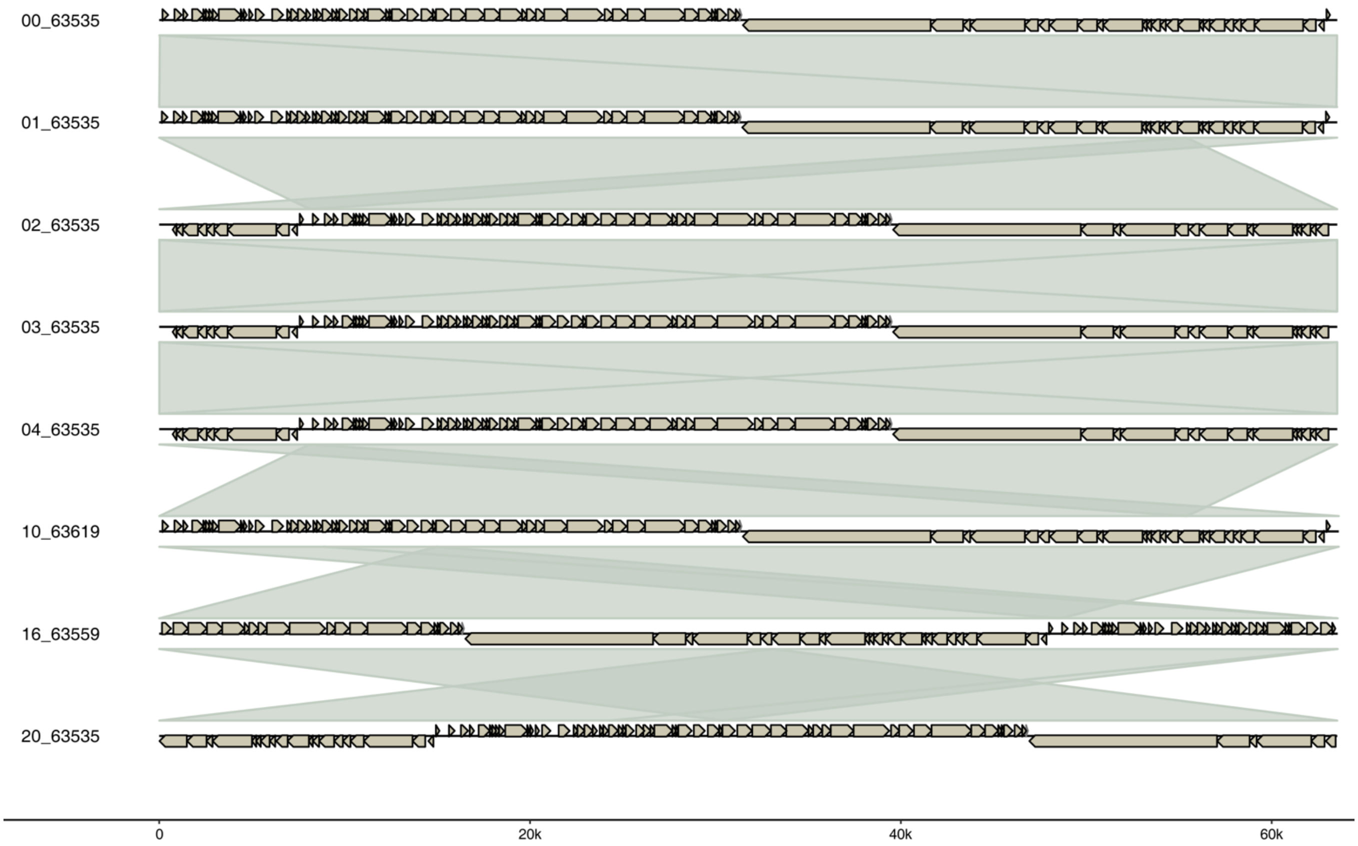

Fig. S22. Genome organization of Theomophage vMAGs.
Theomophage contigs independently assembled from 5_closed samples from transfer 0, 1, 2, 3, 4, 10, 16, and 20. Contigs are circularly permuted relative to each other and have highly consistent length, indicating complete assembly of a bacteriophage genome. Labels indicate transfer number and contig length.

Data S1. Supplementary Tables S1–S11 (provided as an external Excel file)

Table S1. Sample richness estimates.
Mean genus counts of all compost mesocosm metagenomes for different abundance cutoffs (fraction of total sample reads) and filtering strategies. Reads from contigs not annotated at genus level by RAT, for example due to conflicting information from ORFs or incomplete taxonomic annotation of best matches, were either counted as a separate genus, or removed.

Table S2. Coverage statistics of the Theomophage genome across regimes and community types.
Maximum, mean, and standard deviation of the fraction of the Theomophage genome covered by >1 read in samples from each regime (closed, open) and community type (type A, type B).

Table S3. Annotated coding sequences and predicted functions of the Theomophage genome.

Table S4. *Schitoviridae* hallmark gene annotations (HMMScan output).

Table S5. SNPs in Theomophage populations across regimes and community types.
Group indicates in which regime and community type the SNPs were observed. Columns S156. S6 and S34 indicate presence of SNPs in these genotypes.

Table S6. iPHoP host prediction for Theomophage MAG NODE_224_length_63535_cov_5745.934389.

Table S7. Host prediction results from individual tools within iPHoP.

Table S8. CRISPR spacer hits against Theomophage from SpacerDB.

Table S9. CRISPR spacer cluster details from SpacerDB.

Table S10. SpacerDB hits taxonomy summary.

Table S11. tBLASTn hits of S6 cellulase proteins.
ORF94 (YP_007005830.1) and ORF96 (YP_007005832.1). ORF95 resulted in no hits.

Table S12. ENA accessions and sample metadata.
